# Integrating brainstem and cortical functional architectures

**DOI:** 10.1038/s41593-024-01787-0

**Published:** 2024-10-16

**Authors:** Justine Y. Hansen, Simone Cauzzo, Kavita Singh, María Guadalupe García-Gomar, James M. Shine, Marta Bianciardi, Bratislav Misic

**Affiliations:** 1 Montréal Neurological Institute, McGill University, Montréal, Quebec Canada; 2 Brainstem Imaging Laboratory, Department of Radiology, Athinoula A. Martinos Center for Biomedical Imaging, Massachusetts General Hospital and Harvard Medical School, Boston, MA USA; 3 Parkinson’s Disease and Movement Disorders Unit, Center for Rare Neurological Diseases (ERN-RND), University of Padova, Padova, Italy; 4 Multiscale Imaging and Integrative Biophysics Unit, National Institute on Aging, National Institutes of Health, Baltimore, MD USA; 5 Escuela Nacional de Estudios Superiores, Unidad Juriquilla, Universidad Nacional Autónoma de México, Querétaro, Mexico; 6 Brain and Mind Centre, The University of Sydney, Sydney, Australia; 7 Division of Sleep Medicine, Harvard University, Boston, MA USA

**Keywords:** Network models, Neuroscience

## Abstract

The brainstem is a fundamental component of the central nervous system, yet it is typically excluded from in vivo human brain mapping efforts, precluding a complete understanding of how the brainstem influences cortical function. In this study, we used high-resolution 7-Tesla functional magnetic resonance imaging to derive a functional connectome encompassing cortex and 58 brainstem nuclei spanning the midbrain, pons and medulla. We identified a compact set of integrative hubs in the brainstem with widespread connectivity with cerebral cortex. Patterns of connectivity between brainstem and cerebral cortex manifest as neurophysiological oscillatory rhythms, patterns of cognitive functional specialization and the unimodal–transmodal functional hierarchy. This persistent alignment between cortical functional topographies and brainstem nuclei is shaped by the spatial arrangement of multiple neurotransmitter receptors and transporters. We replicated all findings using 3-Tesla data from the same participants. Collectively, this work demonstrates that multiple organizational features of cortical activity can be traced back to the brainstem.

## Abstract (web summary)

Hansen et al. used in vivo functional imaging of the human brainstem and cortex to demonstrate how the brainstem shapes cortical functional architecture, including oscillatory dynamics, cognitive specialization and hierarchical organization.

## Main

The brain is a network of functionally interacting neural populations. Studying the functional architecture of the brain in awake humans is possible with multiple imaging technologies, although these technologies are often biased toward the cortex where signal quality is highest^1^. As a result, key findings about functional activity in the brain—including the presence of functionally specialized brain regions^2^, networks of regions with synchronized neural activity^3,4^ and mechanisms behind higher-order cognitive processes^5^—are primarily limited to the cerebral cortex. An important question is therefore: what role do extracortical structures have in cortical function?

Perhaps the most prominent missing piece of modern in vivo brain network reconstruction is the brainstem. This early evolutionary structure is crucial for survival and consciousness and integrates signals from across the nervous system. In addition, multiple neurotransmitter systems originate in brainstem nuclei and project throughout the cortex, shaping cortical activity^6–8^. In stark contrast to research on cortical function, knowledge about brainstem function comes predominantly from lesion studies or studies in model organisms, and these studies are often limited to specific brainstem nuclei or pathways^9–12^. Exciting recent imaging advances have improved the feasibility of functional imaging in the whole brainstem, including ultra-high-field magnetic resonance imaging (MRI) scanners and extensive brainstem-specific physiological noise reduction pipelines^1,13,14^. Furthermore, recent development of brainstem atlases encompassing multiple nuclei has made it possible to augment the cortical functional connectome with an anatomically comprehensive representation of the brainstem^15^.

In the present study, we investigated how the brainstem’s functional architecture aligns with cortical function by analyzing a high-resolution 7-Tesla resting-state functional MRI (fMRI) dataset in conjunction with a whole-brainstem atlas spanning 58 nuclei across midbrain, pons and medulla. First, we identified hubs of brainstem–cortex connectivity and found that electrophysiological signatures of neural oscillations are reflected by brainstem–cortex functional connectivity (FC). Next, we clustered brainstem nuclei with respect to how they are connected with the cortex and identified communities of brainstem nuclei that subserve familiar cortical functional activation patterns related to memory, social cognition, movement, sensation and emotion. Using positron emission tomography (PET)-estimated brain maps for 18 neurotransmitter receptors and transporters, we found chemoarchitectonic signatures of brainstem–cortex FC. Finally, we demonstrate that the cortical functional hierarchy delineating unimodal (lower-order) and transmodal (higher-order) brain regions reflects patterns of connectivity with the brainstem. Altogether, using simultaneous in vivo human imaging of brainstem and cortical functional activity, this study extends our perspective of cortical function—including dynamics, cognitive function and the unimodal–transmodal cortical functional gradient—to the brainstem, demonstrating how cortical functional architecture consistently reflects brainstem influence.

## Results

**Fig. 1:**
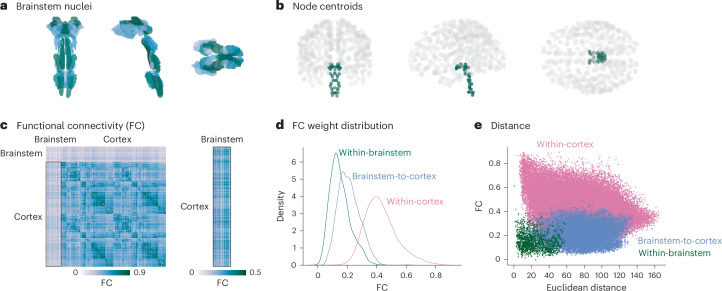
Brainstem–cortex FC. **a**, Coronal (posterior view), saggital and axial view of the thresholded (35%) probabilistic template for all 58 brainstem nuclei in the Brainstem Navigator atlas (https://www.nitrc.org/projects/brainstemnavig/ (ref. ^15^)). **b**, Coronal (posterior view), saggital and axial view of cortical (gray points, *n* = 400) and brainstem (green points, *n* = 58) parcel coordinate centroids. **c**, Left, FC matrix (458 regions × 458 regions). Right, FC matrix between cortex and brainstem (400 cortical regions × 58 brainstem nuclei). **d**, Density distributions of FC within brainstem (green), between brainstem and cortex (blue) and within cortex (pink). **e**, Scatter plot of FC between regions as a function of Euclidean distance between parcel centroids. Within-cortex two-sided Spearman’s *r* = −0.29, *P* ≈ 0; brainstem–cortex two-sided Spearman’s *r* = 0.05, *P* = 8.7 × 10^−16^; within-brainstem two-sided Spearman’s *r* = −0.11, *P* = 3.4 × 10^−6^.

Resting-state fMRI time series in the cortex and brainstem were acquired on a 7-Tesla scanner in 20 unrelated healthy participants (29.5 ± 1.1 years of age, 10 males and 10 females), and replication data were acquired on a 3-Tesla scanner in the same individuals. Brainstem data were processed following established brainstem-specific protocols, and all functional connections were defined based on specified cortical and brainstem seed and target regions (see Methods for details). Cortical regions were defined according to the 400 regions in the Schaefer parcellation^16^, and brainstem nuclei were defined according to the 58 nuclei in the Brainstem Navigator atlas (50 bilateral and eight midline nuclei; Fig. 1a,b; atlas available at https://www.nitrc.org/projects/brainstemnavig)^15^. We validated the brainstem atlas by parcellating PET images of neurotransmitter receptor densities to the brainstem and confirmed that receptors show high density in their associated brainstem nuclei, such as serotonin receptors (5HT_1A_, 5HT_1B_, 5HT_2A_, 5HT_4_ and 5HT_6_) and transporter (5-HTT) in the raphe nuclei, dopamine receptor (D_2_) and transporter (DAT) in the substantia nigra and ventral tegmental area, and noradrenergic norepinephrine transporter (NET) in the locus coeruleus (Supplementary Fig. 1). Next, we confirmed that temporal signal-to-noise ratio (tSNR) in the brainstem, although low, is within the cortical tSNR range (Supplementary Fig. 2a). Finally, we confirmed that smaller brainstem nuclei are not associated with lower tSNR (*r* = −0.45, *P* = 0.0004; Supplementary Fig. 2b,c).

In Fig. 1c, we show FC (Pearson’s correlation between time series) of the brainstem and cortex. Cortical FC shows a familiar network organization and is correlated with FC data from the Human Connectome Project (HCP; Spearman’s *r* = 0.58, *P* ≈ 0 (ref. ^17^)). Interestingly, we found that the brainstem is more functionally connected with the cortex than it is with itself (Fig. 1d; Welch’s two-sided *t*-test *t* = 33.9, *P* < 0.001). Indeed, whereas cortical FC decreases with Euclidean distance^18^, brainstem FC is less affected by distance (*r* = −0.29 and *r* = −0.11, respectively; Fig. 1e). This aligns with the fact that major white matter tracts in the brainstem (for example, medial lemniscus, spinothalamic tract and corticospinal tract^19^) project to regions outside of the brainstem (including cortex, subcortex and spinal cord), which may result in weak FC within the brainstem and stronger FC between brainstem and cortex.

### Brainstem–cortex FC

**Fig. 2:**
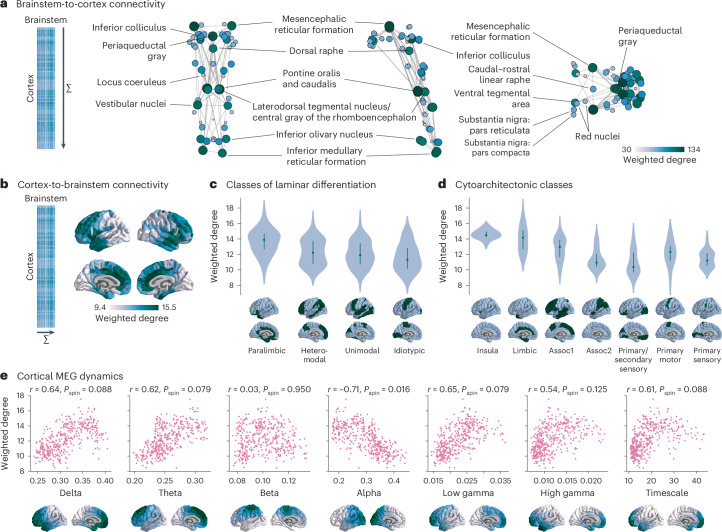
Dominant patterns of brainstem–cortex FC. **a**, Brainstem-to-cortex weighted degree is calculated by summing a brainstem nucleusʼ FC across all cortical regions. Coronal (posterior view), sagittal and axial perspectives of brainstem nuclei are shown. Node size and color reflect weighted degree, and edges are plotted for the 5% strongest functional connections within the brainstem (see Supplementary Fig. 22 for 2.5% and 10% strongest edges). Key brainstem nuclei are labeled. **b**, Cortex-to-brainstem weighted degree was calculated by summing a cortical region’s FC across all brainstem nuclei. Color bar ranges from the 2.5th to 97.5th percentiles of the data. **c**, Cortex-to-brainstem weighted degree binned according to classes of laminar differentiation (groups are significantly different from one another; one-way ANOVA *F* = 18.5, *P* = 2.8 × 10^−11^)^26,94^. Classes: paralimbic (*n* = 61), heteromodal (*n* = 136), unimodal (*n* = 120) and idiotypic (*n* = 3). **d**, Cortex-to-brainstem weighted degree binned according to classes of cytoarchitecture (groups are significantly different from one another; one-way ANOVA *F* = 35.6, *P* = 2.0 × 10^−34^)^95,96^. Classes: insula (*n* = 16), limbic (*n* = 39), association network 1 (*n* = 155), association network 2 (*n* = 77), primary/secondary sensory (*n* = 64), primary motor (*n* = 26) and primary sensory (*n* = 23). Violin plots in **c** and **d** estimate a kernel density on the underlying data, where the underlying data are the weighted degree of each cortical region in the bin. The green point indicates the median, and the vertical line indicates the quartiles of the distribution. **e**, Scatter plots are shown for the correlation between cortex-to-brainstem weighted degree and seven metrics of MEG dynamics: power spectrum distributions for six canonical frequency bands and the intrinsic timescale (temporal memory of a neural element; see Methods for details); each point is a brain region (*n* = 400). Cortical distributions of MEG measures are shown on the brain surface below each plot and are derived from data in the HCP^17^.

The horizontal and vertical stripe patterning of brainstem–cortex FC shown in Fig. 1 indicates that there is a dominant pattern of brainstem–cortex connectivity. Hereafter, we refer to the pattern of connectivity that brainstem nuclei make with the cortex as ‘brainstem-to-cortex’ connectivity and vice versa as ‘cortex-to-brainstem’ connectivity, despite no implication of directionality. The dominant pattern of how brainstem nuclei are connected with the cortex is quantified as the sum of FC across cortical regions (‘weighted degree’; Fig. 2a). Brainstem-to-cortex hubs—brainstem nuclei that are most functionally connected with the cortex—are spatially segregated, in line with the theory that hub placement optimizes the tradeoff between distance and efficient information transfer^20^.

Brainstem-to-cortex hubs in the midbrain include the mesencephalic reticular formation, periaqueductal gray and dorsal raphe^13,14^. Brainstem hubs in the pons include the pontine reticular nuclei, the laterodorsal tegmental nucleus and vestibular nuclei (spanning both pons and medulla). Finally, remaining brainstem hubs in the medulla include the inferior olivary nucleus and inferior medullary reticular formation. We confirmed that the weighted degree pattern is not correlated with tSNR (Spearmanʼs *r* = 0.20, *P* = 0.14). We similarly show the weighted degree pattern in the cortex, which represents how strongly cortical regions are connected with the brainstem (cortex-to-brainstem hubs; Fig. 2b). This pattern follows an anterior–posterior gradient, with the anterior cingulate cortex being a primary hub of cortex-to-brainstem FC. By binning cortical regions’ weighted degree according to their assignment in Mesulam classes of laminar differentiation and von Economo classes of cytoarchitecture, we found that limbic and insular classes demonstrate the greatest brainstem FC, whereas unimodal classes demonstrate the lowest brainstem FC (Fig. 2c,d; Mesulam laminar classes: one-way ANOVA *F* = 18.5, *P* = 2 × 10^−11^; von Economo cytoarchitectonic classes: one-way ANOVA *F* = 35.6, *P* = 2.0 × 10^−34^). Notably, within the limbic and insular cytoarchitectonic classes, the anterior insula and anterior cingulate cortex demonstrate the greatest cortex-to-brainstem FC (Supplementary Fig. 3).

The anterior–posterior cortical gradient of brainstem FC with cortex can be interpreted as a gradient of brainstem influence on cortical neural populations. Therefore, we tested whether this gradient is aligned with more direct measurements of cortical dynamics—that is, neural oscillatory rhythms from electrophysiology. Specifically, we correlated magnetoencephalography (MEG)-derived spectral power distributions for six canonical frequency bands as well as the intrinsic timescale (which can be interpreted as the temporal memory of a neural element) from the HCP with cortex-to-brainstem weighted degree^17,21^. We found that cortex-to-brainstem weighted degree is correlated (*r* > 0.5) with all seven measures of neural oscillatory dynamics, especially alpha power (which survives a spatial autocorrelation-preserving null and multiple comparisons correction; *r* = −0.71, *P*_spin_ = 0.016; Fig. 2e). This demonstrates that cortical dynamics and brainstem input are aligned across multiple temporal scales.

### Brainstem connectivity reflects cognitive ontologies

**Fig. 3:**
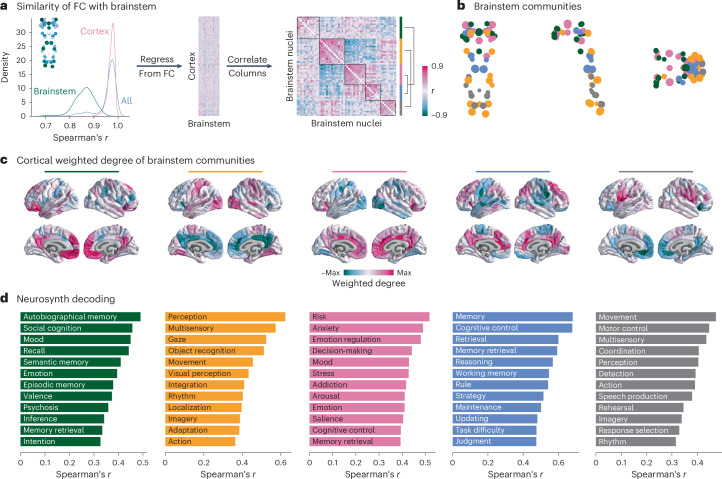
Brainstem communities underlying cortical function. The Louvain community detection algorithm was applied to determine whether brainstem nuclei can be organized into distinct communities that make specific connectivity patterns with the cortex. **a**, Left, for all 458 nodes (400 cortical and 58 brainstem), we correlated (Spearmanʼs *r*) the node’s brainstem FC profile with the weighted degree pattern shown in the inset and in Fig. 2a. The density distribution of Spearman’s *r* for brainstem (green) and cortical (pink) nodes is shown separately as well as together (blue) (median *r* = 0.97). Middle, this brainstem map (weighted degree of brainstem-to-cortex FC) is regressed out of each cortical region’s brainstem FC pattern, resulting in a matrix (400 cortical regions × 58 brainstem nuclei) of FC residuals. Right, correlation matrix representing how similarly (Spearman’s *r*) two brainstem nuclei are functionally connected with the cortex, above and beyond the dominant pattern of connectivity between brainstem and cortex. Brainstem nuclei are ordered according to community affiliation (community colors shown on the right), and communities are outlined within the heatmap. Brackets on the right indicate how communities are joined in coarser community detection solutions. **b**, Community assignments from the Louvain community detection algorithm. Coronal (posterior view), sagittal and axial perspectives of brainstem nuclei are shown. Node size is proportional to weighted degree shown in Fig. 2a. See Table 1 for a list of all brainstem nuclei organized by community affiliation. **c**, Cortical weighted degree patterns were calculated as the sum of a cortical region’s FC with all brainstem nuclei within a specific community and are shown for all five communities. These maps represent how each brainstem community is functionally connected with the cortex. **d**, Each cortical weighted degree pattern in **c** was correlated with 123 cognitive and behavioral meta-analytic activation maps from Neurosynth^22^. Only the top 10% correlations are shown. Correlation coefficients for the full set of Neurosynth terms can be found in Supplementary Fig. 8.

Individual cortical regions and brainstem nuclei are similarly functionally connected with the brainstem following the brainstem weighted degree pattern (Fig. 3a, left; median *r* = 0.97, *r* ∈ [0.90, 1] for cortical regions, *r* ∈ [0.71, 0.94] for brainstem nuclei; see Supplementary Fig. 4 for regional correlation coefficients). To understand how brainstem nuclei are uniquely functionally connected with the cortex, we need to focus on connectivity patterns beyond this dominant pattern. We therefore regressed brainstem weighted degree from each region’s connectivity-with-brainstem profile (Fig. 3a). This resulted in an FC matrix that represents how the brainstem and cortex are connected with one another above and beyond their dominant pattern of connectivity (Fig. 3a, middle). By correlating the regressed cortical connectivity profile of pairs of brainstem nuclei, we constructed a brainstem region × region correlation matrix that represents how similarly any two brainstem nuclei are functionally connected with the cortex (Fig. 3a, right).

This similarity matrix was subjected to the Louvain community detection algorithm at multiple resolution parameters (0.1 < *γ* < 6.0). We found that the brainstem can be divided into a nested hierarchy of communities, with each community representing a group of nuclei that exhibit similar FC patterns with the cortex. We show a stable solution of five approximately equally sized communities at *γ* = 2.8 in the main text (Fig. 3b) as well as two coarser solutions of three (*γ* = 1.9) and four (*γ* = 2.2) communities in the supplement (Supplementary Figs. 5 and 6). Regions within each community are listed in Table 1, and we describe each community in detail below.

**Table 1:** Brainstem communities

Green	Yellow	Pink	Blue	Gray
Median raphe nucleus^*^	Raphe obscurus^*^	Dorsal raphe^*^	Isthmic reticular formation (L)	Raphe magnus^*^
Paramedian raphe nucleus^*^	Raphe pallidus^*^	Caudal–rostral linear raphe^*^	Pontine reticular nucleus: pontis oralis and caudalis (LR)	Parvicellular reticular nucleus: alpha part (L)
Periaqueductal gray^*^	Inferior olivary nucleus (LR)	Ventral tegmental area/ parabrachial pigmented nucleus complex (LR)	Laterodorsal tegmental nucleus/central gray of the rhomboencephalon (LR)	Superior medullary reticular formation (LR)
Substantia nigra: pars reticulata (L)	Lateral parabrachial nucleus (LR)	Medial parabrachial nucleus (R)	Microcellular tegmental nucleus/parabigeminal nucleus (L)	Superior olivary complex (LR)
Substantia nigra: pars compacta (LR)	Inferior medullary reticular formation (LR)	Substantia nigra: pars reticulata (R)	Medial parabrachial nucleus (L)	Viscero-sensory-motor nuclei complex (LR)
Red nucleus: subregion 1 (LR)	Parvicellular reticular nucleus: alpha part (R)	Mesencephalic reticular formation (LR)	Locus coeruleus (LR)	Subcoeruleus (L)
Red nucleus: subregion 2 (R)	Red nucleus: subregion 2 (L)	Cuneiform (LR)		
Pedunculoegmental nuclei (L)	Vestibular nucleus (LR)	Isthmic reticular formation (R)		
Microcellular tegmental nucleus, parabigeminal nucleus (R)	Subcoeruleus (R)	Pedunculotegmental nucleus (R)		
Superior colliculus (LR)	Inferior colliculus (LR)			

Brainstem nuclei within each of the five communities are shown in Fig. 3. Asterisks indicate a midline nucleus. L/R refers to the hemisphere of bilateral nuclei.

How are these brainstem communities connected with the cortex? For each brainstem community, we calculated each cortical region’s total FC (weighted degree; sum of FC across brainstem nuclei) with the brainstem nuclei within that community (Fig. 3c; for variance of FC across brainstem nuclei, see Supplementary Fig. 7b). Cortical weighted degree can be interpreted as a cortical network pattern that is associated with each brainstem community. Next, to determine the functional specialization of each cortical network, we correlated the cortical weighted degree patterns in Fig. 3c with 123 meta-analytic functional activation patterns from Neurosynth (see Methods for details^22^). We show the 12 (10%) most highly correlated Neurosynth keywords in Fig. 3d and show the full list of 123 correlation coefficients per community in Supplementary Fig. 8.

We found a community (yellow) composed of regions throughout the brainstem, including the inferior colliculus, vestibular nuclei and inferior olivary nucleus. This community is most functionally connected with unimodal cortex and associated with sensory perception and movement. A second sensory-related community (gray) exists in the medulla and is composed of regions including the superior olivary complex, the viscero-sensory-motor complex and the raphe magnus. This community is most connected with ventral regions of primary motor and sensory cortex as well as anterior parietal regions, such as the angular and supramarginal gyri, regions that are associated with higher-order motor coordination and speech. Note that the yellow and gray communities are joined in the three-community solution (Supplementary Fig. 5). We also found a community (pink) composed of midbrain regions, including the ventral tegmental area, dorsal and caudal–rostral linear raphe nuclei and mesencephalic reticular formation. This community is most functionally connected with cingulate cortex and is associated with emotion regulation, affect, addiction and arousal. This community’s cortical weighted degree pattern is also most spatially and functionally similar to the dominant weighted degree pattern (Supplementary Fig. 9a,b).

Finally, we found two brainstem communities that are related to higher-order cognitive functions. The first (green) is composed of midbrain regions, including the substantia nigra, red nucleus, superior colliculus and periaqueductal gray. This community is most connected with medial transmodal cortical regions, including the precuneus and frontal pole. The second higher-order cognitive community (blue) is composed of regions in the midbrain and pons, including the locus coeruleus, the laterodorsal tegmental nucleus/central gray of the rhomboencephalon and the pontine reticular nuclei. Both the green and blue communities are functionally connected with transmodal cortex and are associated with memory, but each community is specialized. The green community is most connected with the frontal pole and is associated with autobiographical memory and social cognition; the blue community is connected more broadly to medial and lateral transmodal cortex and is associated with memory retrieval, working memory and cognitive control. Notably, the green community remains isolated in the three-community and four-community solutions, whereas the blue and pink communities are combined (Supplementary Figs. 5 and 6). The three-community solution was also observed using 3-Tesla data (Supplementary Fig. 10). Collectively, these findings demonstrate the striking alignment between cognitive function and brainstem function.

To explore which nuclei may be more functionally flexible, we calculated Spearmanʼs correlation between each brainstem nucleus’ (1) regressed FC with the cortex and (2) the cortical weighted degree pattern of that brainstem nucleus’ assigned community (Supplementary Fig. 7c). Nuclei with the lowest correlations (that is, nuclei that are least represented by their community’s pattern of cortical connectivity) include the median raphe nucleus, the superior colliculi, the pedunculotegmental nuclei, the microcellular tegmental nucleus-parabigeminal nuclei, the subcoeruleus and subregions of the substantia nigra as well as the red nuclei. These nuclei (except the median raphe, which is a midline nucleus) are all bilateral nuclei whose homologs are assigned to different communities. This suggests that these nuclei are involved in both unimodal (sensory-motor) and transmodal (cognition) functions.

### Mapping chemoarchitecture to brainstem communities

**Fig. 4:**
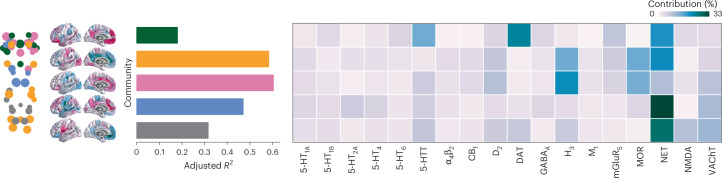
Mapping chemoarchitecture to brainstem communities. For each community (shown on the brainstem plot on the left as well as in Fig. 3b), a multiple linear regression model was fitted between 18 cortical neurotransmitter receptor and transporter density profiles and the community’s cortical weighted degree pattern (shown as surface plots as well as in Fig. 3c). Model fits (adjusted *R*^2^) are shown in the bar plot. Dominance analysis was applied to the independent variables (receptors and transporters) to determine which receptors/transporters were contributing most to the model fit^24^. Percent contribution is shown in the heatmap. Receptor/transporter data were acquired from a PET atlas of neurotransmitter receptor/transporter densities in the human brain^21,23^.

Given that the cortex receives input from multiple neuromodulatory brainstem nuclei, we sought to identify the relationship among neurotransmitter systems, the identified brainstem communities and their cortical projection patterns. We used data from a recent PET atlas of nine neurotransmitter systems in the human brain to estimate cortical distributions of 18 neurotransmitter receptors and transporters^21,23^. Specifically, for each brainstem community, we fitted a multiple linear regression model that predicts the community’s cortical weighted degree profile from receptor and transporter densities (Fig. 4, left). Next, we applied dominance analysis to estimate the relative contribution (‘dominance’) of each receptor and transporter to the overall fit (${R}_{{\rm{adj}}}^{2}$) of the model (Fig. 4, right)^24^.

The NET emerges as a dominant receptor across all communities, peaking in the blue memory community, which includes the primary nucleus for norepinephrine synthesis: the locus coeruleus. The second higher-order cognitive brainstem community (green) is connected with the cortex in a manner that aligns with monoamine transporters, including dopaminergic DAT and serotonergic 5-HTT. Indeed, this community includes the dopaminergic substantia nigra and serotonergic median and paramedian raphe nuclei. NET, DAT and 5-HTT also demonstrate high interactional dominance, defined as the change in *R*^2^ when an independent variable is added to the submodel that includes all other independent variables (Supplementary Fig. 11). This suggests that these transporters share relatively little variance with other variables—that is, they consistently add complementary information to other neurotransmitter systems when predicting cortical weighted degree.

We found that the overall fit (${R}_{{\rm{adj}}}^{2}$) is greatest for the sensory-related (yellow) and affect-related (pink) brainstem communities. In other words, these brainstem nuclei are functionally connected with the cortex in a manner that is more aligned with cortical receptor distributions than other brainstem communities (Fig. 4). The most dominant receptors for both these communities include the histamine receptor H_3_, opioid receptor MOR, NET and dopamine receptor D_2_ and, for the pink community, also serotonin transporter 5-HTT and acetylcholine transporter VAChT. These receptors span multiple neurotransmitter systems and are primarily metabotropic rather than ionotropic. They are also receptors (specifically MOR, H_3_ and 5-HT_1A_) that are most dominant in predicting the cortical weighted degree pattern (Supplementary Fig. 9c). Collectively, these findings highlight the role that multiple transmitter systems play in modulating brainstem–cortex FC.

### Brainstem nuclei delineate unimodal and transmodal cortex

**Fig. 5:**
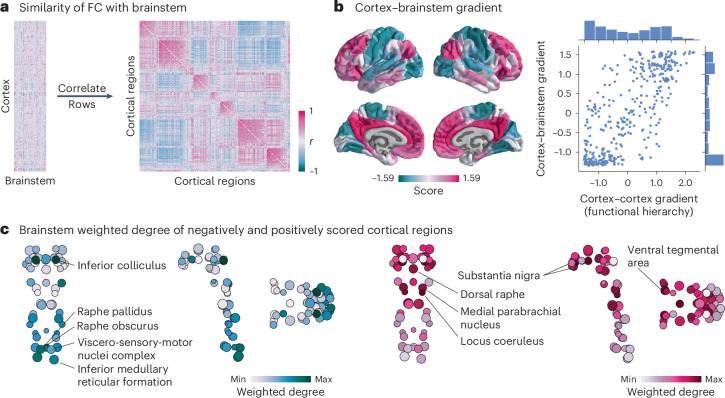
Brainstem nuclei delineate unimodal and transmodal cortical regions. **a**, Left, FC residuals (identical to the matrix shown on the left in Fig. 3a). Right, correlation matrix representing how similarly (Spearman’s *r*) two cortical regions are functionally connected with the brainstem above and beyond the dominant pattern of brainstem–cortex connectivity. **b**, Diffusion map embedding was applied to the matrix shown in **a**. Left, the first gradient of cortex–brainstem FC. Right, correlation between the first gradient of cortex–brainstem connectivity and the first gradient of cortex–cortex FC (also called the cortical functional hierarchy, the unimodal–transmodal axis and the sensory–association axis; *r* = 0.77, *P*_spin_ = 0.0001). Distribution of gradient values are shown for both gradients. **c**, Brainstem weighted degree patterns were calculated as the sum of a brainstem nucleusʼ FC with all negatively (left) or positively (right) scored regions of the cortical gradient shown in **b**. Coronal (posterior view), sagittal and axial perspectives of brainstem nuclei are shown. Node size is proportional to weighted degree shown in Fig. 2a.

Lastly, we asked: if brainstem nuclei demonstrate unique patterns of FC with the cortex, do cortical regions likewise demonstrate unique patterns of FC with the brainstem? Using the regressed functional connectome described above, we correlated the regressed brainstem connectivity profile of pairs of cortical regions to construct a cortical region × region correlation matrix that represents how similarly two cortical regions are functionally connected with the brainstem (Fig. 5a).

We used diffusion map embedding to estimate the first gradient of how similarly cortical regions are connected with the brainstem. Cortical regions with similar scores along this gradient are similarly connected with the brainstem; the greater the difference in gradient scores, the more dissimilar regions are in their brainstem connectivity profiles. This gradient is strongly correlated with the principal functional gradient of cortico–cortical connectivity (also derived using diffusion map embedding; *r* = 0.77, *P*_spin_ = 0.0001; Fig. 5b), which is thought to delineate a hierarchy of cortical function from unimodal (for example, primary regions involved in lower-order functions) to transmodal (for example, association regions involved in higher-order functions) regions^25,26^. We found that this cortical unimodal–transmodal hierarchy also reflects brainstem FC. Interestingly, for the gradient derived from cortical connectivity with the brainstem, most cortical regions are placed at the extremes of the gradient. Indeed, the most stable solution from the Louvain community detection algorithm is one that identifies two prominent communities (one transmodal and one unimodal; Supplementary Fig. 12).

Which brainstem nuclei are more functionally connected with unimodal (negative gradient score) and transmodal (positive gradient score) regions? We calculated the weighted degree of FC from negatively scored and positively scored cortical regions to the brainstem (Fig. 5c). We found that unimodal brain regions are most connected with caudal brainstem nuclei in the medulla, including the inferior medullary reticular formation, the viscero-sensory-motor nuclei complex and the raphe pallidus and obscurus. In addition to these nuclei, the brainstem nucleus with the greatest unimodal connectivity is the inferior colliculus in the midbrain. Likewise, brainstem nuclei most connected with transmodal regions exist in the midbrain and pons, including the ventral tegmental area, the locus coeruleus, the substantia nigra, the dorsal raphe and the medial parabrachial nucleus^27^.

### Replication in the subcortex

Finally, we extended the analyses to other subcortical and diencephalic regions—physically located between the cortex and brainstem and likely mediating their relationship—as a first step toward understanding how the present findings are reflected in subcortical structures (Supplementary Fig. 13a). Specifically, 7-Tesla functional data were also acquired for 14 bilateral non-neocortical FreeSurfer-derived regions (caudate, putamen, pallidum, nucleus accumbens, thalamus, amygdala and hippocampus^28^), eight bilateral Brainstem Navigator diencephalic nuclei (lateral geniculate nucleus (LGN), medial geniculate nucleus (MGN) and subthalamic nuclei subregions 1 and 2 (refs. ^15,29^)) and the hypothalamus^30^. For simplicity, we refer to the FreeSurfer structures as ‘subcortex’ (although the hippocampus is technically allocortex and the thalamus is also part of the diencephalon) and the Brainstem Navigator diencephalic nuclei plus hypothalamus as ‘diencephalon’. Notably, the FreeSurfer-derived regions are large, cytoarchitectonically defined brain regions and do not undergo brainstem-specific pre-processing, whereas the diencephalic nuclei are small nuclei defined from T1 images and do undergo brainstem-specific processing due to their size and proximity to vasculature and cerebrospinal fluid (CSF).

First, we show how strongly each subcortical region and diencephalic nucleus is functionally connected with the brainstem (Supplementary Fig. 13b). Regions with the greatest weighted degree include the thalamus, the hypothalamus and the LGN. Next, we reconstructed the region × region correlation matrix representing how similarly non-neocortical regions are functionally connected with the neocortex, above and beyond the dominant weighted degree pattern of connectivity (Supplementary Fig. 13c). We applied Louvain community detection and found a stable solution of four communities, similar to those described in Fig. 3, with the addition of community affiliations for subcortical and diencephalic regions (regions within each community are listed in Supplementary Table 1). The amygdala, hippocampus, subthalamic nuclei and MGN are grouped with the median raphe nuclei, inferior colliculus and superior olivary complex (gray community). These non-neocortical regions are most functionally connected with primary sensory-motor cortical regions. The nucleus accumbens and hypothalamus are grouped with the periaqueductal gray, cuneiform and superior colliculus (green community). These regions are most functionally connected with ventromedial prefrontal cortex. The caudate and thalamus are grouped with the major neuromodulatory nuclei, including the dorsal raphe, substantia nigra and locus coeruleus (blue community). These regions are most functionally connected with transmodal cortex, including the precuneus, anterior cingulate cortex, angular gyrus and dorsolateral prefrontal cortex. Finally, the putamen and pallidum are grouped with the viscero-sensory-motor complex, vestibular nucleus and inferior olivary nucleus (yellow community). These regions are most functionally connected with ventral primary and secondary motor cortex.

Lastly, we applied diffusion map embedding to the region × region similarity matrix representing how similarly neocortical regions are functionally connected with non-neocortical structures^31^. We found that the first gradient still resembles the unimodal–transmodal gradient (Supplementary Fig. 13d), and we found that the hippocampus, amygdala and MGN have greatest FC with negatively scored (unimodal) brain regions, whereas the caudate, putamen, thalamus and LGN have greatest FC with positively scored (transmodal) brain regions. Altogether, the present findings remain consistent when extended to subcortical regions and diencephalic nuclei.

### Sensitivity and robustness analyses

We conducted three analyses to gauge the sensitivity and robustness of the current findings. First, we ran a split-half resampling analysis where we randomly split the sample of 20 individuals into two groups of 10 (100 repetitions). We correlated the group-averaged functional connectomes, brainstem-to-cortex hub profile and cortex-to-brainstem hub profile between the two groups to assess how much results vary given different samples of participants. We found that FC (0.85 < *r* < 0.95), brainstem-to-cortex hubs (0.90 < *r* < 1) and cortex-to-brainstem hubs (0.7 < *r* < 0.92) are all highly correlated between groups (Supplementary Fig. 14). Second, to ensure that results are robust against alternative parcellations, we repeated the analyses using a 100-region cortical parcellation^16^ and found consistent results for all analyses (Supplementary Fig. 15). Third, because all participants underwent both 7-Tesla and 3-Tesla scanning, we reconstructed their functional connectomes from the 3-Tesla fMRI time series. When comparing the functional connectomes across these two scanning conditions, we found that within-cortex FC was correlated at *r* = 0.40; within-brainstem FC was correlated at *r* = 0.75; and brainstem-to-cortex FC was correlated at *r* = 0.70 (Supplementary Fig. 16). Altogether, these analyses demonstrate that our findings are generalizable across different scanners and processing pipelines.

Finally, we considered the variability of FC across the 20 participants. For every pair of participants (total, 190 pairs), we correlated (Spearmanʼs *r*) their within-cortex FC (mean *r* = 0.40, range (0.12, 0.64)), within-brainstem FC (mean *r* = 0.51, range (0.27, 0.69)) and brainstem–cortex FC (mean *r* = 0.45, range (0.18, 0.63)) (Supplementary Fig. 17a). Consistent with previous literature, FC is sensitive to individual differences^32^. Next, we compared participant brainstem-to-cortex weighted degree (mean *r* = 0.69, range (0.28, 0.88)) and cortex-to-brainstem weighted degree (mean *r* = 0.32, range (−0.03, 0.6)). This shows that brainstem-to-cortex connectivity patterns are more conserved across individuals than cortex-to-brainstem connectivity patterns. Altogether, broad patterns of FC between brainstem and cortex were present in all participants, but individuals are diverse (see Supplementary Fig. 18 for individual FC and weighted degree patterns and Supplementary Fig. 17b–d for standard deviation across participants of FC and weighted degree). Whether individual differences in brainstem–cortex FC are related to individual differences in cognition and behavior is an important question for future work.

## Discussion

In the present study, we used a high-resolution 7-Tesla fMRI dataset in conjunction with a comprehensive brainstem atlas of 58 nuclei to investigate how cortical function reflects brainstem function. We identified a compact set of integrative hubs in the brainstem with strong FC with the cortex. We found that multiple cortical phenomena, including oscillatory rhythms, cognitive function and the unimodal–transmodal hierarchy, can be traced back to specific FC with the brainstem.

In vivo functional imaging of the human brainstem has long eluded the neuroimaging field due to the challenges of imaging this constellation of deep structures, resulting in a vacuum of knowledge about awake human brainstem activity^33^. In the last decade, substantial progress has been made to acquire a robust functional signal in the brainstem. Extensive research on the sources of fMRI signal in the brainstem and the necessity of noise correction has improved the acquisition and interpretability of signals in deep brain structures^34–36^. In 2015, Bianciardi et al.^15^ began developing an in vivo neuroimaging template of human brainstem nuclei that facilitated the standardization of whole-brainstem functional imaging. Later, in 2022, Singh et al.^14^ and Cauzzo et al.^13^ reported the resting state functional connectomes of functionally defined arousal and motor^14^ and autonomic, limbic, pain and sensory^13^ brainstem nuclei to the rest of the brain. In the present study, we joined these connectomes into a single dataset of whole-brainstem to whole-cortex FC to ask: what can in vivo human whole-brainstem functional activity tell us about cortical function?

First, we located the regions in the brainstem that are most functionally connected with the cortex. Although there exists a rich literature of hubs in cortex^37^, little is known about the hubs in the brainstem^13,14,27^. We identified a set of integrative brainstem hubs that are located throughout the midbrain, pons and medulla. Brainstem-to-cortex hubs are functionally diverse, with some thought to be primarily involved in motor functions (for example, inferior olivary nucleus (motor coordination), pontine nuclei (movement) and vestibular nuclei (balance)), some that are associated with specific neurotransmitter systems (for example, dorsal raphe (serotonin) and laterodorsal tegmental nucleus (acetylcholine)^8^) and some that have been linked to multiple functions (for example, mesencephalic and inferior medullar reticular formation and periaqueductal gray). Surprisingly, the locus coeruleus is not identified as a hub, despite its known widespread projections throughout the cortex and its role in information integration^38^.

Likewise, we demonstrate that cortical regions follow an anterior–posterior gradient with respect to their FC strength to the brainstem, with the largest cortex-to-brainstem hubs existing in anterior cingulate cortex. Previous studies reported greater diffusion-weighted MRI-derived structural connectivity from anterior cortex to brainstem^39,40^. Similarly, transcriptomic analysis of human von Economo neurons, whose cell bodies are restricted to Layer V of the anterior cortex, showed that these bipolar neurons express transcriptional factors associated with long-range projections to the brainstem^41,42^. In other words, this gradient of cortex–brainstem FC likely reflects the underlying synaptic connectivity between brainstem and cortex. Interestingly, the anterior–posterior gradient of cortex-to-brainstem FC parallels an anterior–posterior gradient within the insula (Supplementary Fig. 3). This is noteworthy because the insula (although binned into a single cytoarchitectonic class in Fig. 2d) is functionally diverse^43^. We found that the insular regions with greatest brainstem FC are the ventral anterior insular cortex (a region related to visceromotor control) and the dorsal anterior insular cortex (related to attention)^44,45^. Middle and posterior insular regions related to sensory functions, such as olfaction, gustation and interoception, are less functionally connected with the brainstem^46^.

In addition, we found a close correspondence between cortex-to-brainstem hubs and MEG-derived alpha power. Although cortical rhythms have been extensively studied, subcortical and brainstem rhythms are difficult to measure because of electrophysiological signal decay across larger distances^47^. One theory of the functional role of the alpha rhythm is that alpha represents inhibitory input from thalamus to cortex—that is, a ‘closed thalamic gate’^48^ (but see ref. ^49^ for an extensive discussion on different theories of alpha oscillation function). This is in line with our finding that greater cortex–brainstem synchrony occurs in cortical regions with low alpha power. We also found that cortex-to-brainstem weighted degree is positively correlated with cortical power distributions of slow frequencies (for example, delta and theta), which may reflect their role in cognition^50^. Altogether, our work suggests that brainstem connectivity informs the grammar of ongoing cortical dynamics, prompting future work to test the relationship between cortical and brainstem rhythms^51,52^.

Functional imaging has been used to demonstrate networks of cortical regions that co-activate both during specific tasks and at rest^3,4^. Recent studies that explored extracortical structures of the central nervous system demonstrated that cortical networks also co-activate with specific cerebellar regions^31,53,54^, spinal cord segments^55^ and specific brainstem nuclei^27,56^. Rather than imposing cortically defined patterns of functional activation on the brainstem, we asked whether brainstem nuclei share similar connection patterns with the cortex and, if so, what are the cortical networks of brainstem connectivity. We found that the brainstem can be organized into hierarchical communities of nuclei with similar cortical connectivity. These communities establish functional links between brainstem nuclei that were previously unknown and can likely be observed only in living humans where it is possible to record neural activity simultaneously from the brainstem and cortex. Furthermore, each brainstem community is connected with familiar cortical functional networks underlying cognition, memory, sensation, movement and emotion. A small number of bilateral brainstem nuclei are assigned to separate communities with both unimodal and transmodal characteristics, potentially suggesting that these nuclei are functionally flexible (Table 1). Indeed, these are also nuclei that demonstrate lower correlations with their respective community’s cortical weighted degree patterns (Supplementary Fig. 7c). Collectively, these findings indicate that the brainstem has widespread involvement in multiple cognitive and behavioral functions^27,57^.

Each community is associated with multiple psychological functions. A natural explanation is that this is due to their specific chemoarchitectural makeup. Namely, the brainstem is made up of multiple neuromodulatory systems that project throughout the cortex, tuning large-scale synchronization of neuronal populations and emergent functions^7,8^. A major neuromodulatory system that projects throughout the brain is the noradrenergic system^58^. We found that NET is closely aligned with each brainstem community’s associated cortical activation pattern and that this relationship is strongest in the community (blue) that houses the noradrenergic locus coeruleus (Fig. 3). Furthermore, this community is related to memory, cognitive control and retrieval, all integrative functions thought to be modulated by the norepinephrine system^59^. The primacy of NET across all five brainstem communities is perhaps counterintuitive prima facie, given that the locus coeruleus is not identified as a hub. However, previous work speculated that the locus coeruleus’ integrative properties emerge only during specific behavioral contexts^60^. This suggests that the locus coeruleus, and perhaps brainstem hubs in general, may be state dependent and temporally variable. Therefore, the dominance of NET may suggest that its spatial patterning makes NET present and available to promote brainstem–cortex synchrony across multiple cognitive contexts. Indeed, NET’s presence regardless of brain state is consistent with the fact that NET functions to end norepinephrine action in the synapse^61^ and is a non-specific transporter involved in the re-uptake of other monoamines^62^. In addition, noradrenergic projections from the locus coeruleus may specifically target cortical regions and networks depending on the cognitive context. The state dependency of the locus coeruleus can be tested in the future with brainstem-optimized fMRI experiments during tasks^63,64^. A second, non-exclusive explanation is that the NET tracer ([^11^C]MRB) suffers from decreased signal-to-noise due to a scan duration longer than the half-life of ^11^C (ref. ^65^). Ultimately, more work is necessary to disentangle the relationship among the locus coeruleus, norepinephrine and NET regarding brainstem–cortex FC.

Finally, we found that cortical regions are connected with the brainstem following a well-known and frequently studied cortical gradient: the sensory–association axis^25,26,31,66,67^. The sensory–association axis, or unimodal–transmodal functional hierarchy, describes a gradient of cortical function from lower-order to higher-order processes. This gradient is aligned with cortical expansion across ontogeny and phylogeny^68^, becomes more polarized with development^69^ and becomes less polarized with pathological progression^70^. Notably, the sensory–association axis is generally observed from and interpreted in light of cortical processing and cortico–cortical connectivity. Here, we found that the poles of the sensory–association axis demonstrate distinct connectivity patterns with the brainstem. This may indicate that functional inputs from the brainstem anchor the polar extremes of the cortical hierarchy (that is, primary and association cortex), whereas cortico–cortical connectivity patterns fill in the gradual shift from lower-order to higher-order cortical functions. In other words, the hierarchy of cortical function may emerge from connectivity patterns with the brainstem, bringing to light the influence that extracortical structures can have on cortico–cortical connectivity. How the brainstem is involved in gradient changes across development, healthy aging^12^ and pathology is an exciting question for future research.

Thus far, we have primarily situated our findings within the human neuroimaging literature. However, brainstem connectivity and function have long been studied in non-human model organisms, such as mouse, rat, cat and macaque. These studies typically focus on a specific brainstem nucleus or class of nuclei and use anterograde and retrograde viral tracers to map efferent and afferent neuronal projections ex vivo. Tract-tracing studies consistently report dense projections between prefrontal cortex and the brainstem^71,72^, consistent with our finding that anterior cortex is a hub of brainstem connectivity. Porrino and Goldman-Rakic^71^ reported that the majority of brainstem projections to anterior cortex originate in the ventral tegmental area, the substantia nigra, the dorsal raphe, the locus coeruleus and the medial parabrachial nucleus. These five nuclei are all identified as brainstem nuclei with high FC with transmodal cortex (Fig. 5c). Indeed, although the locus coeruleus makes widespread projections throughout the cortex, projections to prefrontal regions produce a greater release of norepinephrine than those to motor cortex in rats, further supporting the notion that the locus coeruleus is functionally linked with transmodal cortex^73^. Regarding serotonergic raphe nuclei, previous studies reported a dichotomy between projection patterns of rostral and caudal raphe nuclei, such that rostral nuclei (for example, dorsal raphe and median raphe) tend to project rostrally to the cortex, whereas caudal nuclei (for example, raphe pallidus and raphe obscurus) tend to project caudally into the spinal cord or to visceral and somatic motor nuclei in the reticular formation of the brainstem^74,75^. In particular, most serotonergic raphe neurons that innervate the cortex are sent from the dorsal raphe^76^. We observed the same dichotomy between rostral and caudal raphe nuclei in terms of connectivity to transmodal and unimodal cortex, respectively, and we also identified the dorsal raphe as a hub of brainstem–cortex FC. Altogether, ex vivo anatomical studies in non-human species support the present human in vivo fMRI findings.

Why should the brainstem be more anatomically and functionally connected with anterior cortical regions? One hypothesis is that this pattern of connectivity is related to the brain’s allostasis: how the brain efficiently maintains energy regulation in the body^77,78^. The cortical regions with the greatest brainstem connectivity are also agranular and dysgranular regions involved in both visceromotor control (motor control of the internal body) and interoception (sensation of the internal body)^79^—that is, the ventral anterior insula and the anterior cingulate cortex. Visceromotor control and interoception are crucial for maintaining the body’s energy expenditure (allostasis): the brain anticipates the body’s metabolic needs via interoception, attempts to meet these needs via visceromotor control and then tunes in on whether needs were met via interoception once more^80^. These cortical regions are also fundamental to many functional processes (that is, are ‘domain-general’), including emotion, memory, reward and cognitive control^81^. We found that brainstem nuclei involved in visceromotor control and interoception (periaqueductal gray, parabrachial nuclei and viscero-sensory-motor nuclei complex) are similarly domain-general: rather than being clustered together in the same functional module (Fig. 3), these nuclei are spread across all five identified communities related to memory, sensory-motor functions and emotion. Furthermore, the parabrachial nuclei tend to be less well aligned with their assigned community’s cortical projection patterns, again suggesting domain-general function. This is in contrast to skeletomotor (motor control of skeletal muscles) and exteroceptive (sensation of the external world) brain regions (for example, ‘unimodal’ or primary sensory regions), which tend to receive little brainstem input and are typically domain specific. Collectively, we speculate that brainstem–cortex FC may reflect the brainstem’s involvement in allostasis.

Although the primary focus of this work is the brainstem–cortex functional relationship, an important group of structures likely plays a prominent role in brainstem–cortex connectivity: the subcortex. We found that the thalamus is a dominant hub of brainstem connectivity, which supports the notion that most brainstem projections to the cortex travel through the thalamus^82,83^. Brainstem nuclei clustered with the thalamus are overwhelmingly neuromodulatory (Supplementary Table 1)^82,84^ and are functionally connected with the cortex in transmodal regions, suggesting a link among higher-order cognitive processes, the thalamus and neuromodulatory brainstem nuclei. In addition, we found that the link between ventromedial limbic regions and brainstem nuclei, such as the periaqueductal gray, may be mediated by the hypothalamus. Anatomical tract-tracing studies in the macaque demonstrated that the hypothalamus and orbitofrontal and medial limbic regions are connected and that these connections are likely involved in autonomic responses to emotional stimuli^85^. Finally, we found that brainstem nuclei related to motor control, such as the inferior olivary nucleus, the vestibular nuclei and the viscero-sensory-motor nuclei complex, are clustered with subcortical regions also heavily involved in motor control (for example, putamen and globus pallidus)^86,87^. Indeed, these brainstem nuclei and subcortical regions are most functionally connected with ventral and secondary motor cortices. Altogether, by using in vivo fMRI, we can observe functionally relevant relationships among specific brainstem nuclei, subcortical structures and cortical regions^31^.

In the present study, we extended our umwelt of in vivo cortical functional networks to the brainstem and found that multiple cortical phenomena are reflected by brainstem–cortex FC. This opens doors for many future applications of brainstem FC. For example, multiple pathological markers, such as α-synuclein in Parkinson’s disease, are thought to emerge from brainstem dysfunction before spreading throughout the cortex^88,89^. Brainstem FC patterns may generate more accurate models of disease propagation and aberrant dynamics, giving rise to potentially actionable brainstem targets^90^. Brainstem FC may also facilitate the development of better computational models of ongoing dynamics^91^. Although the present work extends the study of in vivo human cortical function to the brainstem, it is increasingly possible to integrate not only brainstem function but also the structure and function of the cerebellum, subcortex and spinal cord into a single wiring diagram of the complete human central nervous system^39,40,92,93^.

We close with some important methodological considerations. First, brainstem nuclei are notoriously difficult to image, given their deep location, proximity to vasculature and CSF, irregular shape and small size. This brainstem dataset underwent extensive and optimized physiological noise correction and validation of the defined nuclei, but brainstem imaging is an active area of research, and best practices continue to be refined. For example, warping from individual space to template space can induce minor overlap of brainstem nuclei borders. In addition, medial temporal and orbitofrontal areas suffer from some dropout and lower tSNR, and brainstem signals demonstrate low residual correlation with signal from the 4th ventricle. Second, the temporal resolution of the 7-Tesla fMRI was minimized at 2.5 s. This was necessary given the number of slices and spatial resolution required to reconstruct small brainstem nuclei. Third, only 20 healthy participants were included in this study. Although we replicated the findings using 3-Tesla scans in the same participants and performed a split-half resampling analysis, future work is necessary to validate our findings in larger datasets. Fourth, the optimal brainstem registration may result in suboptimal cortical registration, although we found that within-cortex FC was correlated with FC from an independent dataset. Fifth, the present report mainly considers group-averaged FC despite the variability in FC across individuals (Supplementary Figs. 17 and 18). As acquisition and processing protocols for the brainstem become established, datasets with more individuals will make it possible to test whether individual variability in brainstem FC is predictive of individual differences in cognition.

In summary, we mapped the functional architecture of brainstem–cortex connectivity. We found that the functional architecture of the brainstem is an ever-present leitmotif of cortical function. The present work takes advantage of advances in modern brain imaging, extending the scope of inquiry to structures that were previously inaccessible and ultimately leading to a more complete understanding of the brain.

## Methods

### fMRI data acquisition

fMRI data in the brainstem were collected, pre-processed and originally presented in ref. ^13^ and ref. ^14^. The study protocol was approved by the Massachusetts General Hospital institutional review board. No statistical methods were used to predetermine sample size. After providing written informed consent in accordance with the Declaration of Helsinki, 20 unrelated healthy participants (age 29.5 ± 1.1 years, 10 males and 10 females) participated in two eyes-closed resting-state 7-Tesla and 3-Tesla MRI sessions (Magnetom and Connectom, respectively; Siemens Healthineers). No participants were excluded in the present report, and participants were compensated $35 USD per hour of participation. During the 7-Tesla session, three runs of 10 min were acquired, whereas a single run of 9 min was acquired at 3 Tesla. Notably, brainstem-specific custom protocols were developed for the 7-Tesla MRI acquisition and processing, which we describe below, whereas conventional sequences were used for the 3-Tesla MRI acquisition. Complete details for all acquisition and processing parameters are detailed in full in both ref. ^13^ and ref. ^14^.

In brief, a custom-built 32-channel receive coil and volume transmit coil was used at 7 Tesla, and a custom-built 64-channel receive coil and volume transmit coil was used at 3 Tesla. For each participant, three runs of 7-Tesla functional gradient-echo echo-planar images were acquired with the following parameters: isotropic voxel size = 1.1 mm, matrix size = 180 × 240, GRAPPA factor = 3, nominal echo spacing 0.82 ms, bandwidth = 1,488 Hz/Px, number of slices = 123, slice orientation = sagittal, slice acquisition order = interleaved, echo time (TE) = 32 ms, repetition time (TR) = 2.5 s, flip angle = 75°, simultaneous multi-slice factor = 3, number of repetitions = 210, phase-encoding direction = anterior–posterior and acquisition time = 10 min, 7 s. Between the three fMRI runs, the awake state of participants was verified verbally. Foam pads were used to minimize head motion, and earplugs were provided. To account for physiology-related signal fluctuations, timing of cardiac and respiratory cycles was recorded via piezoelectric finger pulse sensor (ADInstruments) and piezoelectric respiratory bellow (UFI), respectively. To correct for geometric distortion, a 2.0-mm isotropic resolution fieldmap was acquired. Finally, an anatomical T1-weighted multi-echo MEMPRAGE image was acquired for each participant, with the following parameters: isotropic voxel size = 1 mm, TR = 2.53 s, TEs = 1.69, 3.5, 5.3, 7.2 ms, inversion time = 1.5 s, flip angle = 7°, field of view = 256 × 256 × 176 mm^3^, bandwidth = 650 Hz/Px, GRAPPA factor = 3, slice orientation = sagittal, slice acquisition order = anterior–posterior and acquisition time$=4{\prime} 28$ (ref. ^13^).

### fMRI data pre-processing

Physiological noise correction was done in each resting-state fMRI run using a custom-built MATLAB function of RETROICOR^97^ adapted to the slice acquisition sequence. Functional images were then slice-time corrected, reoriented to standard orientation and co-registered to the MEMPRAGE image. Co-registration was implemented in AFNI using a two-step procedure made of an affine co-registration and a boundary-based (edge enhancing) nonlinear co-registration^98^. Quality of the fMRI co-registration to MNI template space was evaluated for each individual to ensure that brainstem nuclei as defined by the template aligned with individual anatomy. Next, six rigid-body motion time series nuisance regressors, a regressor describing respiratory volume per unit time convolved with a respiration response function^99^, a regressor describing heart rate convolved with a cardiac response function^100^ and five regressors modeling the signal in CSF, extracted using principal component analysis (PCA) on a mask of the brainstem-surrounding ventricles (specifically, the lower part of the 3rd ventricle, cerebral aqueduct and 4th ventricle), were regressed from the fMRI time series. Therefore, by design, the time series of brainstem nuclei are not correlated with the signal from the surrounding ventricles^101,102^. See Supplementary Fig. 19 for correlations between brainstem nucleus signal and 4th ventricle signal as well as between brainstem nucleus signal and PC1 of brainstem-surrounding CSF signal. Cleaned data were scaled to percent signal change by dividing by the temporal signal mean, multiplying by 100 and band-pass filtering between 0.01 Hz and 0.1 Hz. Finally, any residual temporal mean was removed, and the three runs were concatenated.

### Brainstem nuclei segmentation

Brainstem nuclei were defined according to the Brainstem Navigator, a previously developed probabilistic atlas of in vivo brainstem (*n* = 58 (eight midline and 50 bilateral)) and diencephalic (*n* = 8 (four bilateral)) nuclei^15^. The segmentation of the hypothalamus (*n* = 1 midline region) was from ref. ^30^. Nuclei are defined in MNI152NLin6Asym 1-mm^3^ space (matrix size: 182 × 218 × 182). We provide an overview of how the brainstem nuclei were segmented; the original descriptions can be found in refs. ^15,29,103–106^. In brief, 12 (six males and six females, age 28 ± 1 years) healthy participants underwent 7-Tesla MRI imaging where a T2-weighted image and a diffusion-weighted image were acquired. The diffusion-weighted image was used to compute diffusion fractional anisotropy (FA) at every voxel. The T2-weighted and FA images displayed high contrast for brainstem nuclei and were used to label the nuclei. First, in ref. ^15^, three raphe nuclei (median raphe, dorsal raphe and raphe magnus), the periaqueductal gray, the substantia nigra and the red nuclei were segmented. The segmentation was done per participant semi-automatically, by clustering either the FA or the T2-weighted image (depending on the nucleus). Clusters were identified as nuclei, and, in some cases, when a cluster contained multiple nuclei, nuclei were separated from one another manually, using prior knowledge about nucleus anatomy (see Fig. 2 in ref. ^15^ for an overview of this procedure). This results in a per-participant binary mask for each nucleus. Labels were aligned to MNI152 template space, averaged across individuals and converted to a probability where 100% indicates that the voxel is always identified as the specific nucleus across the 12 individuals. For all nuclei in the Brainstem Navigator atlas, the semi-automatic and manual segmentations ensured that no nuclei overlapped; however, the group-averaged probabilistic templates make it possible that nuclei overlap slightly (degree of overlap depends on the threshold applied to the probabilistic templates).

Later, in ref. ^106^, the mesopontine tegmental nuclei were segmented (cuneiform, pedunculotegmental nuclei, oral pontine reticular nuclei, paramedian raphe and caudal-linear raphe). Here, the same semi-automatic procedure was applied, but two additional validations were applied: (1) nuclei were also segmented manually by a neurosurgeon who used the T2-weighted and FA image contrasts as well as mesopontine tegmental anatomical landmarks; and (2) segmented nuclei were compared to a postmortem histologically defined atlas^107^. All subsequent nuclei segmented in later studies were delineated manually using multiple experts. The final labels (per individual) were defined as the intersection of the segmentations provided by the independent experts, and the probabilistic templates were generated by averaging the segmentations across participants. Segmentations were validated against the Paxinos atlas^107^. Specifically, in ref. ^29^, the inferior and superior colliculi and superior olivary complex were segmented (as well as the MGN and LGN, which are part of the thalamus). Singh et al.^105^ defined the lateral and medial parabrachial nuclei, the vestibular nuclei complex and the medullary viscero-sensory-motor nuclei. The lateral and medial parabrachial nuclei were additionally validated using histological evaluation from a postmortem human brainstem specimen. Singh et al.^104^ segmented the mesencephalic reticular formation, isthmic reticular formation, microcellular tegmental nucleus, ventral tegmental area and the caudal–rostral linear raphe nucleus complex. Finally, García-Gomar et al.^103^ segmented the raphe obscurus, raphe pallidus, locus coeruleus, subcoeruleus, laterodorsal tegmental nucleus-central gray of the rhombencephalon, inferior and superior medullary reticular formation and the pontine reticular nucleus (oral/caudal part).

### Functional network reconstruction

To construct a two-dimensional functional connectome for each participant, we used the 400-region Schaefer atlas in the cortex^16^ and the 58-nucleus Brainstem Navigator atlas in the brainstem (https://www.nitrc.org/projects/brainstemnavig/) to define seed and target regions^15,29,103–106^. In all analyses, we thresholded the probabilistic atlas at 35%. Note that this threshold did result in some overlapping voxels for bordering nuclei. Specifically, in the 8,334 voxels labeled as part of a brainstem nucleus, 7,922 (95%) were labeled only once, whereas 405 (4.8%) were labeled twice, and seven (0.08%) were labeled three times. No voxels were labeled more than three times. Because brainstem nuclei vary in size (quantified as the number of voxels within each region), with the smallest nucleus (median raphe) at 7 mm^3^ and the largest (periaqueductal gray) at 450 mm^3^, we confirmed that parcel size does not reflect tSNR (Supplementary Fig. 2a for tSNR, Supplementary Fig. 2b for parcel size (nucleus volume) and Supplementary Fig. 2c for their correlation). tSNR was calculated as the mean of the time series divided by the standard deviation (before demeaning the time series in the pre-processing steps outlined above), averaged across participants and parcellated to the defined cortical and brainstem regions. Finally, FC was defined as Pearson’s correlation between time series for every pair of brain regions (458 total). The group-averaged connectome was calculated as the mean across individual connectomes. Analyses were repeated using a 100-region Schaefer atlas as part of the robustness analysis. Analyses were also repeated after including an additional eight diencephalic nuclei from the Brainstem Navigator atlas, the hypothalamus^30^ and 14 FreeSurfer subcortical structures^28^ (subcortical surfaces plotted using the enigmatoolbox^108^). The final FC matrix was compared with a standard 3-Tesla FC matrix from the HCP (326 unrelated participants, age range 22–35 years, 145 males, S900 release), downloaded from https://github.com/netneurolab/hansen_many_networks (ref. ^109^).

In the main text, we do not threshold FC. However, because lower tSNR in the brainstem results in noisier and less reliable signal and, therefore, likely also smaller estimates of FC^81^, we repeated the analyses using a thresholded version of the group-averaged FC matrix (Supplementary Fig. 20). To generate the thresholded matrix, we applied a Fisher transform to the individual-specific FC matrices (Pearson’s correlation coefficients)^13^. Then, we implemented a two-tailed one-sample *t*-test to obtain a group statistic. The threshold was defined at *P* < 0.0005 (Bonferroni corrected for multiple comparisons). We set all edges in the group-averaged FC matrix (Pearson’s correlation coefficients; shown in Fig. 1c) where *P* > 0.0005 to 0, resulting in an FC matrix with 53.65% remaining connections.

Although all analyses in the main text were conducted using group-averaged, parcellated FC (Fig. 1c), we show the standard deviation and coefficient of variation (standard deviation normalized by mean) of FC across all 20 participants in Supplementary Fig. 17b. We also show standard deviation of brainstem-to-cortex weighted degree (mean shown in Fig. 2a) and cortex-to-brainstem weighted degree (mean shown in Fig. 2b) across participants in the same figure (Supplementary Fig. 17c,d). Lastly, we show these three properties (FC, brainstem weighted degree and cortical weighted degree) for every participant in Supplementary Fig. 18. For statistical tests that assume normal distribution, data distributions were assumed to be normal, but this was not formally tested.

### Laminar and cytoarchitectonic classes

We stratified cortex-to-brainstem hubs according to Mesulam classes of laminar differentiation as well as von Economo cytoarchitectonic classes (Fig. 2c,d). The Mesulam classes of laminar differentiation represent four levels of laminar differentiation (idiotypic, unimodal, heteromodal and paralimbic) derived from ref. ^110^ that integrate neuroanatomical, electrophysiological and behavioral studies in humans and non-human primates. Assignments of laminar differentiation to the surface were done manually by Paquola et al.^94^ and parcellated to the Schaefer 400 parcellation^16^ Note that the Mesulam classes of laminar differentiation describe both anatomical arrangement as well as cortical connectivity patterns. An alternative and more direct mapping of laminar differentiation is that proposed by Barbas et al.^111^ (although there does not currently exist a version of the Barbas classification for the human surface). Likewise, the von Economo cytoarchitectonic classes were manually assigned to cortical regions by Vértes et al.^96^, by visual comparison of von Economo and Koskinas’s parcellation and anatomical landmarks^112^ The original von Economo and Koskinas classification includes only five classes; ‘limbic’ (including entorhinal, retrosplenial, presubicular and cingulate cortices) and ‘insular’ cortices were added as two additional classes. Cytoarchitectonic classes were then projected to the surface in ref. ^94^ and parcellated to the Schaefer 400 parcellation^16^. Brain plots of all classes are shown in Fig. 2.

### MEG data acquisition and pre-processing

Six-minute resting-state eyes-open MEG time series were acquired from the HCP S1200 release for 33 unrelated participants (age range, 22–35 years, 17 males)^17,113^. Complete MEG acquisition protocols can be found in the HCP S1200 Release Manual. For each participant, we computed the power spectrum at the vertex level across six different frequency bands—delta (2–4 Hz), theta (5–7 Hz), alpha (8–12 Hz), beta (15–29 Hz), low gamma (30–59 Hz) and high gamma (60–90 Hz)—using the open-source software Brainstorm^114^. The pre-processing was performed by applying notch filters at 60, 120, 180, 240 and 300 Hz and was followed by a high-pass filter at 0.3 Hz to remove slow-wave and DC-offset artifacts. Pre-processed sensor-level data were used to obtain a source estimation on HCP’s fsLR4k cortex surface for each participant. Head models were computed using overlapping spheres, and the data and noise covariance matrices were estimated from the resting-state MEG and noise recordings. Brainstorm’s linearly constrained minimum variance (LCMV) beamformers method was applied to obtain the source activity for each participant. Welch’s method was then applied to estimate power spectrum density for the source-level data, using overlapping windows of length 4 s with 50% overlap. Average power at each frequency band was then calculated for each vertex (that is, source). Source-level power data were then parcellated into 400 and 100 cortical regions for each frequency band, according to the Schaefer atlas^16^. Intrinsic timescale of the MEG signal was estimated using spectral parameterization with the FOOOF (fitting oscillations and one over *f*) toolbox^115^, via the method developed by Gao et al.^116^. Power spectral density and intrinsic timescale maps were first calculated and analyzed in refs. ^117,118^. All pre-processed brain maps were downloaded directly from neuromaps^21^.

### Community detection

To identify communities of brainstem nodes that are similarly connected with the cortex, we applied the Louvain community detection algorithm^119^. Because both brainstem and cortex are connected with the brainstem following a dominant pattern (Fig. 2a), we first regressed this weighted degree pattern from every node’s connectivity-to-brainstem profile (Fig. 3a). The residuals represent the degree to which nodes are connected with one another above and beyond this dominant pattern of connectivity. Second, we constructed a brainstem region × brainstem region similarity matrix by correlating the cortical connectivity profiles of every pair of brainstem nodes. This similarity matrix was subjected to the Louvain algorithm, which maximizes positive correlations within communities and negative correlations between communities.

Specifically, brainstem nodes were assigned to communities in a manner that maximizes the quality function

1 $$\begin{array}{l}Q(\gamma )=\frac{1}{{m}^{+}}[{w}_{ij}^{+}-\gamma {p}_{ij}^{+}]\delta ({\sigma }_{i},{\sigma }_{j})\\\qquad\quad\,-\frac{1}{{m}^{+}+{m}^{-}}\sum\limits_{ij}[{w}_{ij}^{+}-\delta {p}_{ij}^{-}]\delta ({\sigma }_{i},{\sigma }_{j})\end{array}$$

where ${w}_{ij}^{+}$ is the network with only positive correlations and likewise for ${w}_{ij}^{-}$ and negative correlations. The term ${p}_{ij}^{\pm }=({s}_{i}^{\pm }{s}_{j}^{\pm })/(2{m}^{\pm })$ represents the null model: the expected density of connections between nodes *i* and *j*, where ${s}_{i}^{\pm }={\sum }_{j}{w}_{ij}^{\pm }$ and ${m}^{\pm }={\sum }_{i,\;j\ > \ i}{w}_{ij}^{\pm }$. The variable *σ*_*i*_ is the community assignment of node *i*, and *δ*(*σ*_*i*_, *σ*_*j*_) is the Kronecker function and is equal to 1 when *σ*_*i*_ = *σ*_*j*_ and 0 otherwise. The resolution parameter, *γ*, scales the relative importance of the null model, wither greater *γ* (*γ* > 1) making it more difficult to detect large communities. In other words, as *γ* increases, increasingly fine network partitions, and more communities, are identified. We tested 60 values of *γ*, from *γ* = 0.1 to *γ* = 6.0, in increments of 0.1. At each *γ*, we repeated the algorithm 250 times and constructed a consensus partition, following the procedure recommended in ref. ^120^.

For each *γ*, the similarity of the clustering solution across the 250 partitions was calculated as the *z*-score of the Rand index. Consensus partitions are considered better quality (that is, more stable) when the mean of the *z*-scored Rand index is high and the variance is low. We show the mean and variance of the *z*-scored Rand index across all *γ*, as well as the number of communities identified, in Supplementary Fig. 21. We show the community solution at *γ* = 2.8 in the main text because it identifies approximately equally sized communities (Fig. 3). We also show solutions at *γ* = 2.2 and *γ* = 1.9 in the supplement (Supplementary Figs. 5 and 6).

### Neurosynth

Probabilistic measures of the association between voxels and cognitive processes were obtained from Neurosynth, a meta-analytic tool that synthesizes results from more than 14,000 published fMRI studies by searching for high-frequency keywords (such as ‘pain’ and ‘attention’) that are published alongside fMRI voxel coordinates (https://github.com/neurosynth/neurosynth; using the volumetric association test maps^22^). This measure of association is the probability that a given cognitive process is reported in the study if there is activation observed at a given voxel. Although more than 1,000 cognitive processes are reported in Neurosynth, we focused primarily on cognitive function and, therefore, limit the terms of interest to cognitive and behavioral terms. These terms were selected from the Cognitive Atlas, a public ontology of cognitive science^121^, which includes a comprehensive list of neurocognitive processes. We used 123 terms, ranging from umbrella terms (‘attention’, ‘emotion’) to specific cognitive processes (‘visual attention’, ‘episodic memory’), behaviors (‘eating’, ‘sleep’) and emotional states (‘fear’, ‘anxiety’). The coordinates reported by Neurosynth were parcellated according to the Schaefer atlas and *z*-scored^16^. The full list of cognitive processes is shown in Supplementary Fig. 8.

### Neurotransmitter receptors and transporters

PET-derived receptor density data were collated by Hansen et al.^23^ and downloaded from neuromaps (https://github.com/netneurolab/neuromaps (ref. ^21^)) for 18 neurotransmitter receptors and transporters across nine neurotransmitter systems. These include dopamine (D_2_ (refs. ^122–126^), DAT (ref. ^127^)), norepinephrine (NET (refs. ^128–131^)), serotonin (5-HT_1A_ (ref. ^132^), 5-HT_1B_ (refs. ^133–139^), 5-HT_2A_ (ref. ^132^), 5-HT_4_ (ref. ^132^), 5-HT_6_ (refs. ^140,141^), 5-HTT (ref. ^132^)), acetylcholine (α_4_β_2_ (refs. ^142,143^), M1 (ref. ^144^), VAChT (ref. ^145^)), glutamate (mGluR_5_ (ref. ^146^)), GABA (GABA_A_ (ref. ^147^)), histamine (H_3_ (ref. ^148^)), cannabinoid (CB_1_ (refs. ^149–152^)) and opioid (MOR (ref. ^153^)). Methodological details about each tracer can be found in Supplementary Table 2. Volumetric PET images were parcellated according to both the Schaefer atlas and the Brainstem Navigator atlas^15,16^.

### Dominance analysis

Dominance analysis seeks to determine the relative contribution (‘dominance’) of each independent variable to the overall fit (adjusted *R*^2^) of the multiple linear regression model (https://github.com/dominance-analysis/dominance-analysis (ref. ^24^)). This is done by fitting the same regression model on every combination of input variables (2^*p*^ − 1 submodels for a model with *p* input variables). Total dominance is defined as the average of the relative increase in *R*^2^ when adding a single input variable of interest to a submodel, across all 2^*p*^ − 1 submodels. The sum of the dominance of all input variables is equal to the total adjusted *R*^2^ of the complete model, making total dominance an intuitive method that partitions the total effect size across predictors. Therefore, unlike other methods of assessing predictor importance, such as methods based on regression coefficients or univariate correlations, dominance analysis accounts for predictor–predictor interactions and is interpretable.

Interactional dominance is the increase in *R*^2^ when adding an independent variable to the submodel that already includes all other independent variables. (As total dominance is defined as the average change in *R*^2^ across all submodels, interactional dominance is one term in this average.) A variable with high interactional dominance contributes more to the linear model in the presence of all other variables and vice versa for low interactional dominance. Therefore, interactional dominance indirectly reflects the shared variance between a variable and the other variables in the model. Total dominance and interactional dominance are normalized by the total fit (${R}_{{\rm{adj}}}^{2}$) of the model, to make dominance fully comparable both within and across models. The normalized total dominance (percent contribution) is plotted in the heatmap in Fig. 4, and the normalized interactional dominance (percent contribution) is plotted in the heatmap in Supplementary Fig. 11.

### Spatial null model

Spatial autocorrelation-preserving permutation tests were used to assess statistical significance of associations across brain regions, termed ‘spin tests’^154–156^. We created a surface-based representation of the parcellation on the FreeSurfer fsaverage surface using files from the Connectome Mapper toolkit (https://github.com/LTS5/cmp). We used the spherical projection of the fsaverage surface to define spatial coordinates for each parcel by selecting the coordinates of the vertex closest to the center of the mass of each parcel. These parcel coordinates were then randomly rotated, and original parcels were reassigned the value of the closest rotated parcel according to the Hungarian algorithm (10,000 repetitions)^157^. The procedure was performed at the parcel resolution rather than the vertex resolution to avoid upsampling the data and for each hemisphere separately. To compute a *P* value, we counted the number of times the null test statistic (for example, Spearman’s correlation with rotated brain map) was more extreme than the empirical test statistic (for example, Spearman’s correlation with original brain map), after de-meaning. Finally, we added 1 to the numerator and normalized by the number of rotations plus 1 (10,001) such that the smallest possible *P* value was 0.0001. All statistical tests using the spin test were two-sided.

### Reporting summary

Further information on research design is available in the Nature Portfolio Reporting Summary linked to this article.

## Online content

Any methods, additional references, Nature Portfolio reporting summaries, source data, extended data, supplementary information, acknowledgements, peer review information; details of author contributions and competing interests; and statements of data and code availability are available at 10.1038/s41593-024-01787-0.

## Supplementary information

Supplementary Information Supplementary Figs. 1–22 and Supplementary Tables 1 and 2

Reporting Summary

## Data Availability

All pre-processed data used to perform the analyses are available at https://github.com/netneurolab/hansen_brainstemfc. MEG power spectral data and neurotransmitter receptor/transporter data are available in neuromaps (https://github.com/netneurolab/neuromaps). Neurosynth data are available at https://neurosynth.org/, and the Cognitive Atlas is available at https://www.cognitiveatlas.org/.

## Code availability

All code used to perform the analyses is available at https://github.com/netneurolab/hansen_brainstemfc. Python version 3.8.10 was used for running scripts.
