# Supplementary material for: Integrating brainstem and cortical functional architectures
Source: Nat Neurosci. 2024 Oct 16;27(12):2500–11. doi: 10.1038/s41593-024-01787-0 (PMC11614745; doi:10.1038/s41593-024-01787-0)
Supplement: Supplementary file 1 — Supplementary Figs. 1–22 and Supplementary Tables 1 and 2 [file 41593_2024_1787_MOESM1_ESM.pdf]

nature neuroscience

Supplementary information https://doi.org/10.1038/s41593-024-01787-0

# Integrating brainstem and cortical functional architectures

In the format provided by the authors and unedited

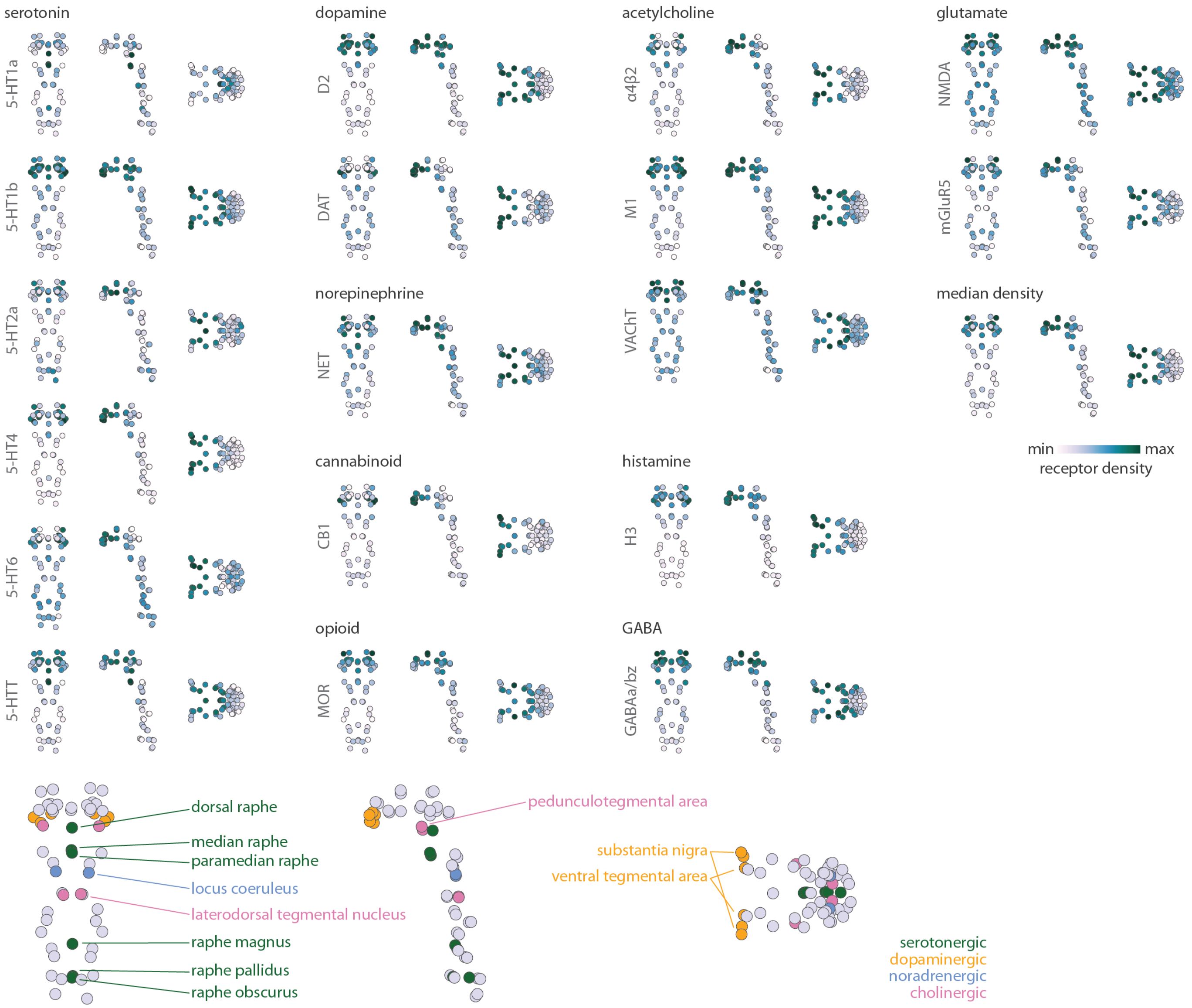


Figure S1. **Neurotransmitter receptor and transporter densities in the brainstem |** 18 PET-derived neurotransmitter receptor and transporter density profiles are shown in the brainstem, as well as the median density across all 18 maps. Coronal (posterior view), sagittal, and axial perspectives of brainstem nuclei are shown. A legend of neuromodulatory nuclei in the brainstem is shown in the bottom row.

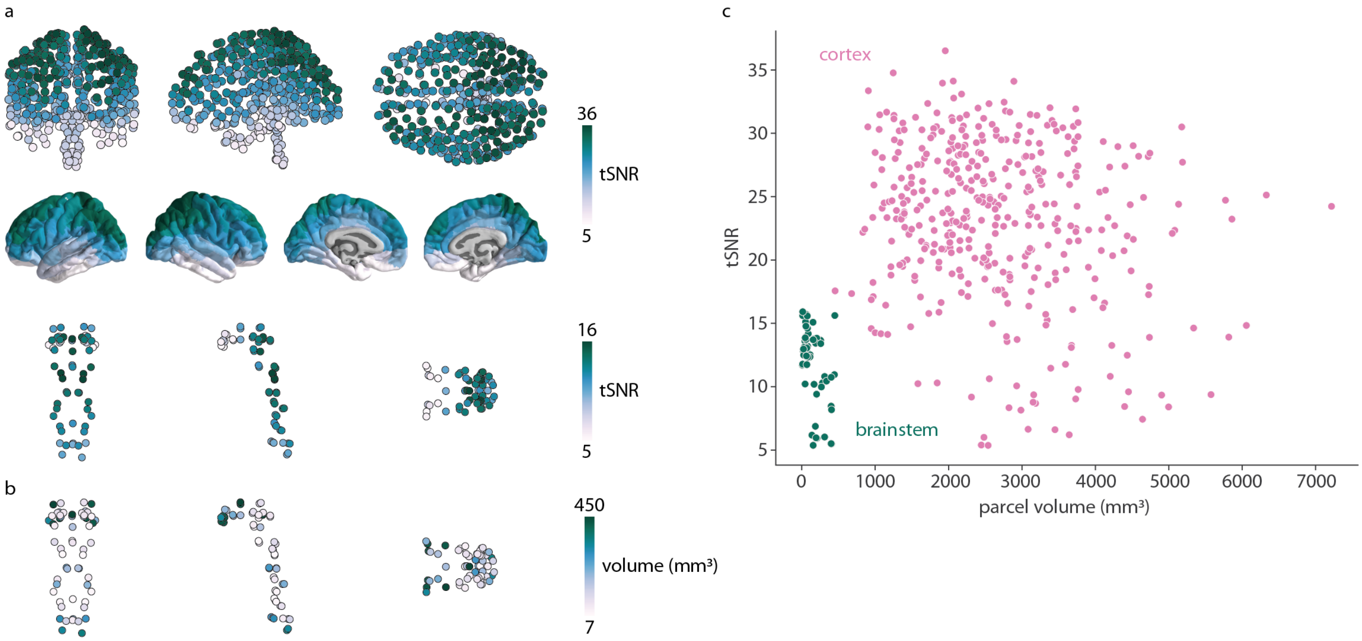


Figure S2. **Temporal signal-to-noise ratio and parcel size |** (a) Temporal signal-to-noise ratio (tSNR) is calculated as the ratio of the mean of a region's time-series to its standard deviation (prior to demeaning the time-series in the preprocessing pipeline). tSNR is shown for the cortex and brainstem together (top), just the cortical surface (middle) and just the brainstem (bottom). Cortical tSNR $\in [5.37, 36.51]$, brainstem tSNR $\in [5.38, 15.92]$. (b) Parcel volume in mm$^3$ is shown for each brainstem nucleus. (c) Scatter plot showing the relationship between parcel volume and tSNR of brainstem (green; two-sided Spearman's $r = -0.45$, $p = 0.0004$) and cortical (pink; two-sided Spearman's $r = -0.15$, $p = 0.003$) regions.

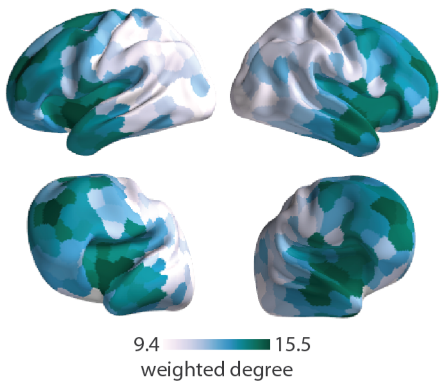


Figure S3. **Cortex-to-brainstem weighted degree |** Here we replot cortex-to-brainstem weighted degree (Fig. 2b) on the fsLR inflated surface lateral view (top), and the fsLR very inflated surface anterior-lateral view (bottom), which makes the insula more visible.

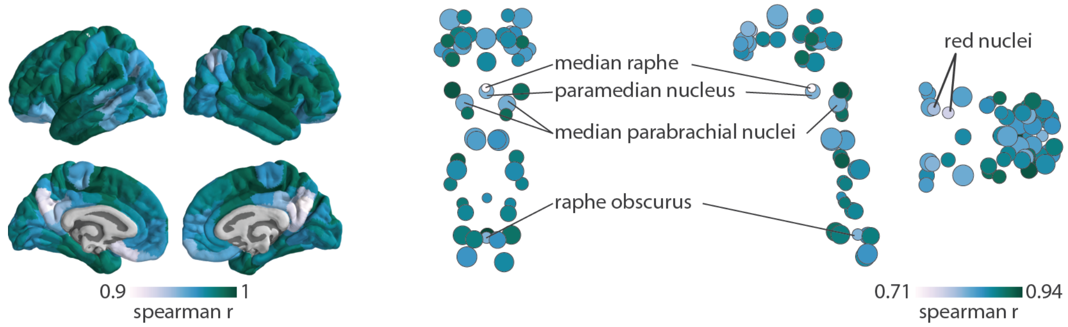


Figure S4. **Regional variation in alignment with brainstem weighted degree |** For each cortical and brainstem region, we correlate their brainstem FC with the brainstem weighted degree pattern shown in Fig. 2a. Cortical regions with the lowest correlations include the orbitofrontal cortex and the precuneus. Brainstem nuclei with the smallest correlations are the raphe nuclei (median raphe, raphe obscurus), the red nuclei, the paramedian nucleus, and the medial parabrachial nuclei. Coronal (posterior view), sagittal, and axial perspectives of brainstem nuclei are shown. Note that the median raphe node was arranged in front of the paramedian nucleus for visualization purposes.

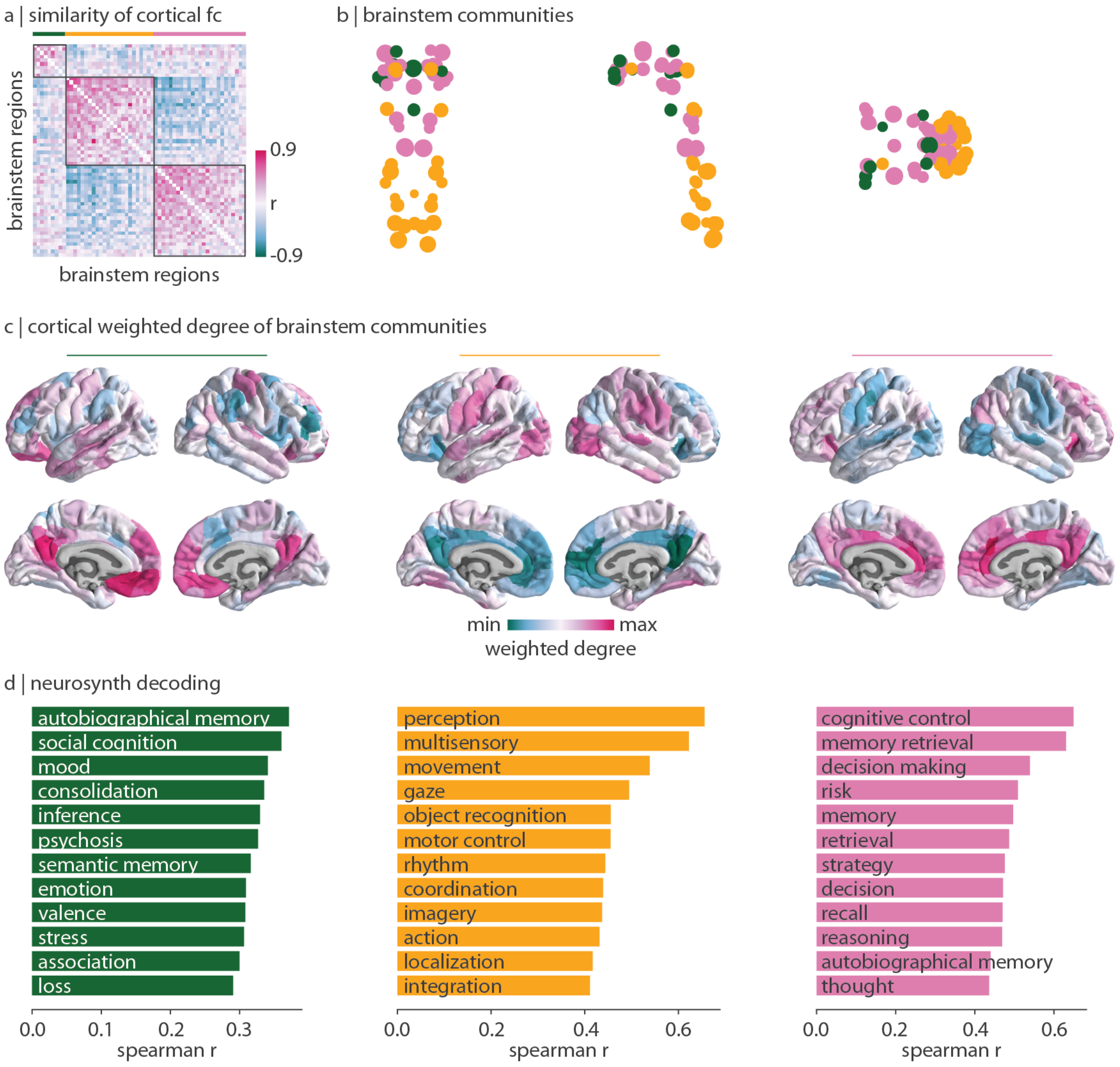


Figure S5. **Brainstem communities when $\gamma = 1.9$ |** The Louvain community detection algorithm was repeated for $\gamma = 1.9$ which identified three stable communities.

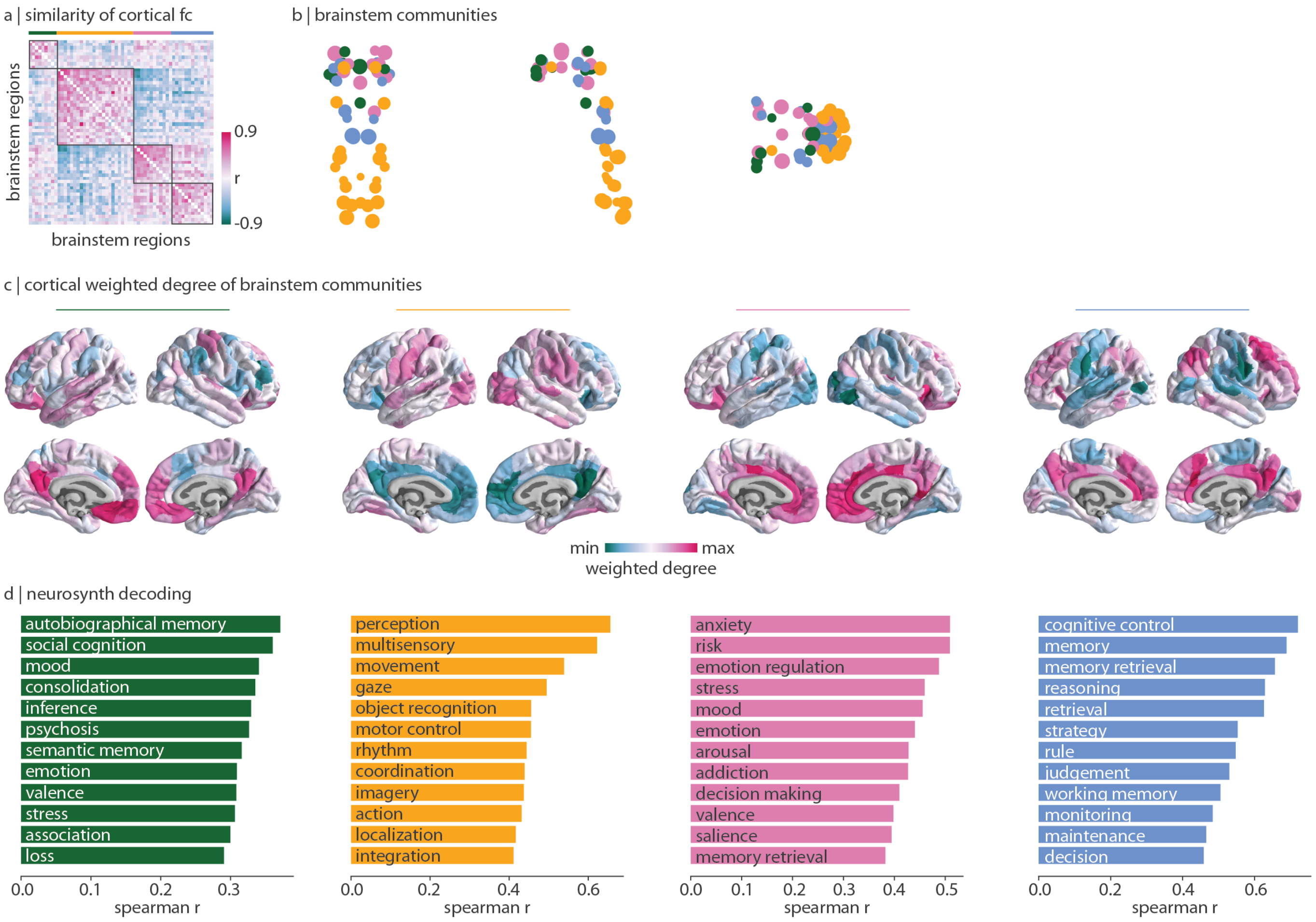


Figure S6. **Brainstem communities when** $\gamma = 2.2$ **|** The Louvain community detection algorithm was repeated for $\gamma = 2.2$ which identified four stable communities.

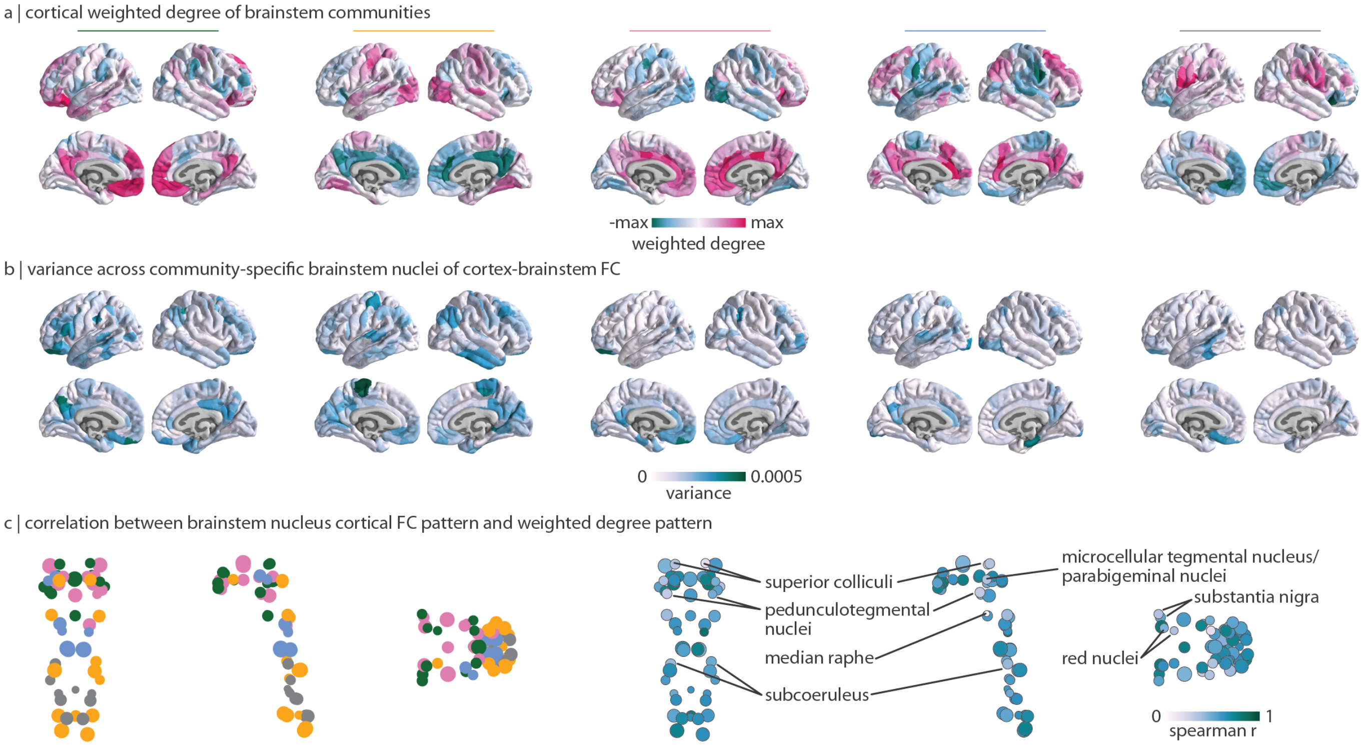


Figure S7. **Variability in brainstem-cortex functional connectivity in communities |** (a) For each brainstem community, we plot each cortical region's weighted degree (sum of FC across brainstem nuclei within a community; identical to Fig. 3 and directly proportional to the mean). (b) For each brainstem community, we plot each cortical region's variance of FC across brainstem nuclei within a community. (c) Left: brainstem community assignments (identical to Fig. 3b). Right: for each brainstem nucleus, we correlate its (regressed) cortical FC pattern with the cortical weighted degree pattern of its associated community (shown in panel (a)).

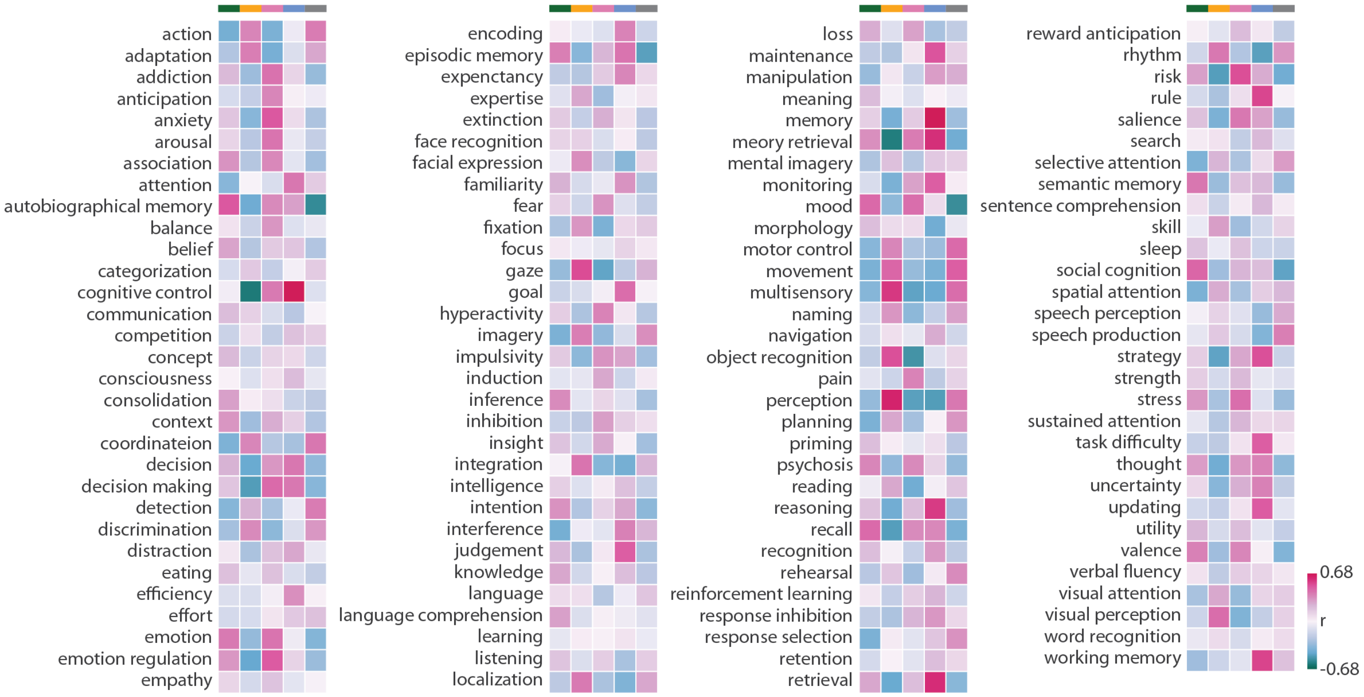


Figure S8. **Neurosynth profiles for each brainstem community |** For each of the five brainstem communities (indicated by the colours at the top of each column, which correspond to the colours in Fig. 3, we show the Spearman correlation coefficients for all 123 Neurosynth terms used in the analysis.

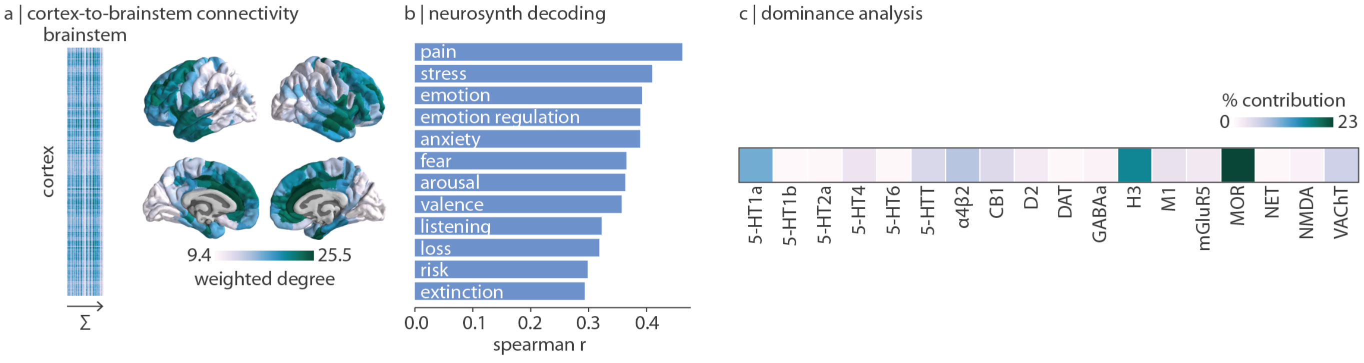


Figure S9. **Psychological and chemoarchitectonic signatures of the dominant cortical weighted degree pattern |** In Fig. 3 and Fig. 4 we consider psychological and chemoarchitectonic signatures of brainstem-cortex functional connectivity, above and beyond the dominant brainstem-cortex patterns of connectivity. Here we repeat these analyses using the cortical weighted degree pattern itself. (a) The cortical weighted degree pattern (representing cortex-to-brainstem functional connectivity and identical to Fig. 2b). (b) The top 12 (10%) Spearman correlation coefficients between the weighted degree pattern in (a) and 123 Neurosynth functional association maps. (c) A multiple linear regression model was fit using the weighted degree pattern in (a) as the dependent variable and PET-derived densities for 18 neurotransmitters and transporters as the independent variables ($R^2_{adj} = 0.57$). Dominance analysis was applied to the independent variables; shown is the percent contribution (total dominance normalized by $R^2_{adj}$) of each receptor/transporter.

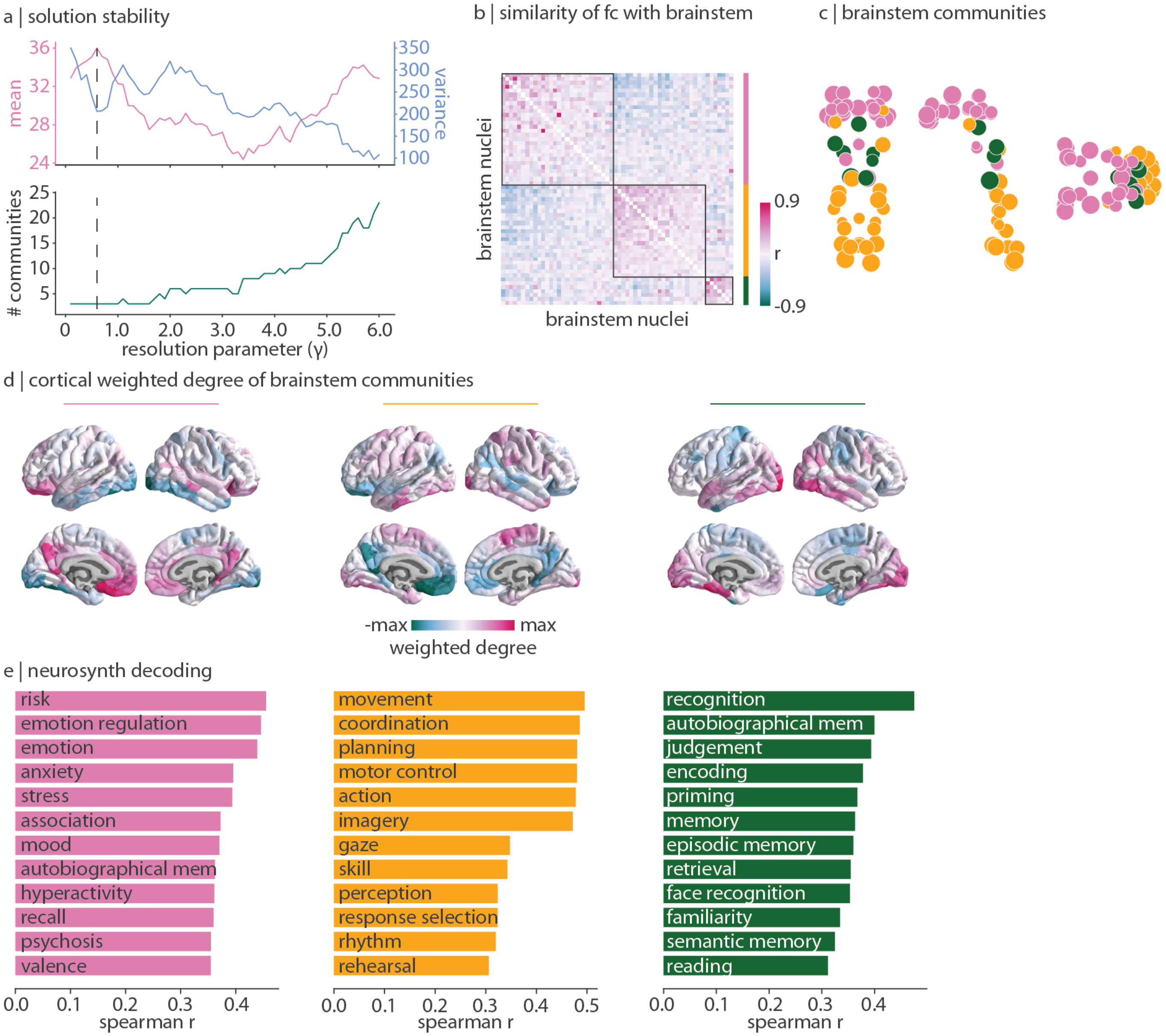


Figure S10. **Brainstem communities at 3T |** The Louvain community detection was repeated for 3T functional connectivity data. (a) Top: mean and variance of the z-scored Rand index across 250 repetitions of the Louvain algorithm at each resolution parameter $\gamma \in [0.1, 6.0]$. Bottom: the number of communities identified for each $\gamma$. The dashed vertical line exists at $\gamma = 0.6$. (b) Brainstem nuclei $\times$ nuclei correlation matrix representing how similarly brainstem nuclei are functionally connected with the cortex (after regressing out brainstem-to-cortex weighted degree, and at 3T). Nuclei are ordered according to the three communities identified at $\gamma = 0.6$. (c) Community affiliation for each brainstem nucleus. Coronal (posterior view), sagittal, and axial perspectives of brainstem nuclei are shown. (d) Cortical weighted degree of the three communities. (e) Each cortical weighted degree pattern in panel (c) was correlated with 123 cognitive and behavioural meta-analytic maps from Neurosynth[22]. Only the top 10% correlations are shown.

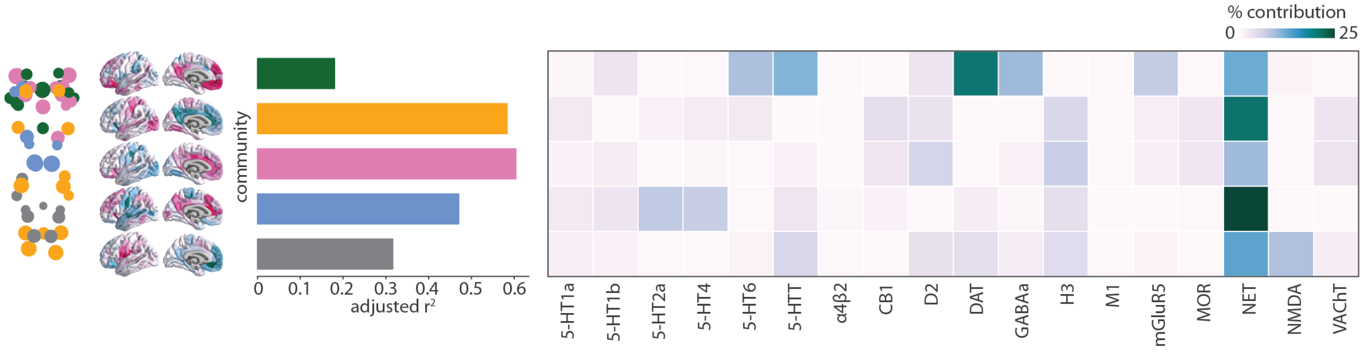


Figure S11. **Interactional dominance** | For each community (shown on the brainstem plot on the left as well as in Fig. 3, a multiple linear regression model was fit between 18 cortical neurotransmitter receptor and transporter density profiles and the community's cortical weighted degree pattern (shown as surface plots, as well as in Fig. 3c). Model fits (adjusted $R^2_{\text{adj}}$) are shown in the bar plot, identical to in Fig. 4. The heatmap shows interactional dominance, defined as the change in $R^2_{\text{adj}}$ when an independent variable is added to the submodel where all other independent variables are present, normalized by the total $R^2_{\text{adj}}$ of the model (one model per row).

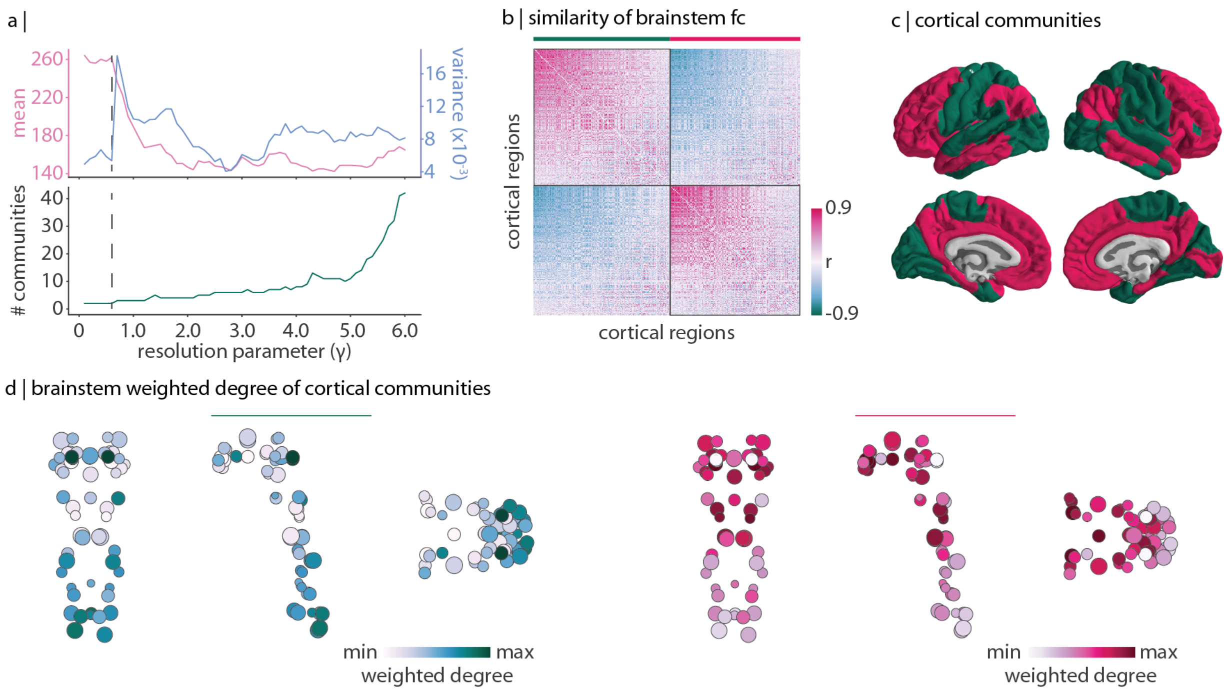


Figure S12. **Cortical communities of brainstem functional connectivity** | The Louvain community detection algorithm was applied to a correlation matrix representing how similarly (Spearman's $r$) two cortical regions are functionally connected with the brainstem above and beyond the dominant pattern of brainstem connectivity. (a) Top: mean and variance of the z-scored Rand index across 250 repetitions of the Louvain algorithm at each resolution parameter $\gamma \in [0.1, 6.0]$. Bottom: number of communities identified for each $\gamma$. The dashed vertical line exists at $\gamma = 0.6$. (b) Cortical region $\times$ region correlation matrix representing how similarly cortical regions are functionally connected with the brainstem. Regions are ordered according to the two communities identified at $\gamma = 0.6$. (c) Community affiliations for each cortical region. (d) Brainstem weighted degree of the green (left, unimodal) community and the red (right, transmodal) community. Specifically, for each brainstem nucleus, we sum its regressed functional connectivity with all cortical regions in the green/red community.

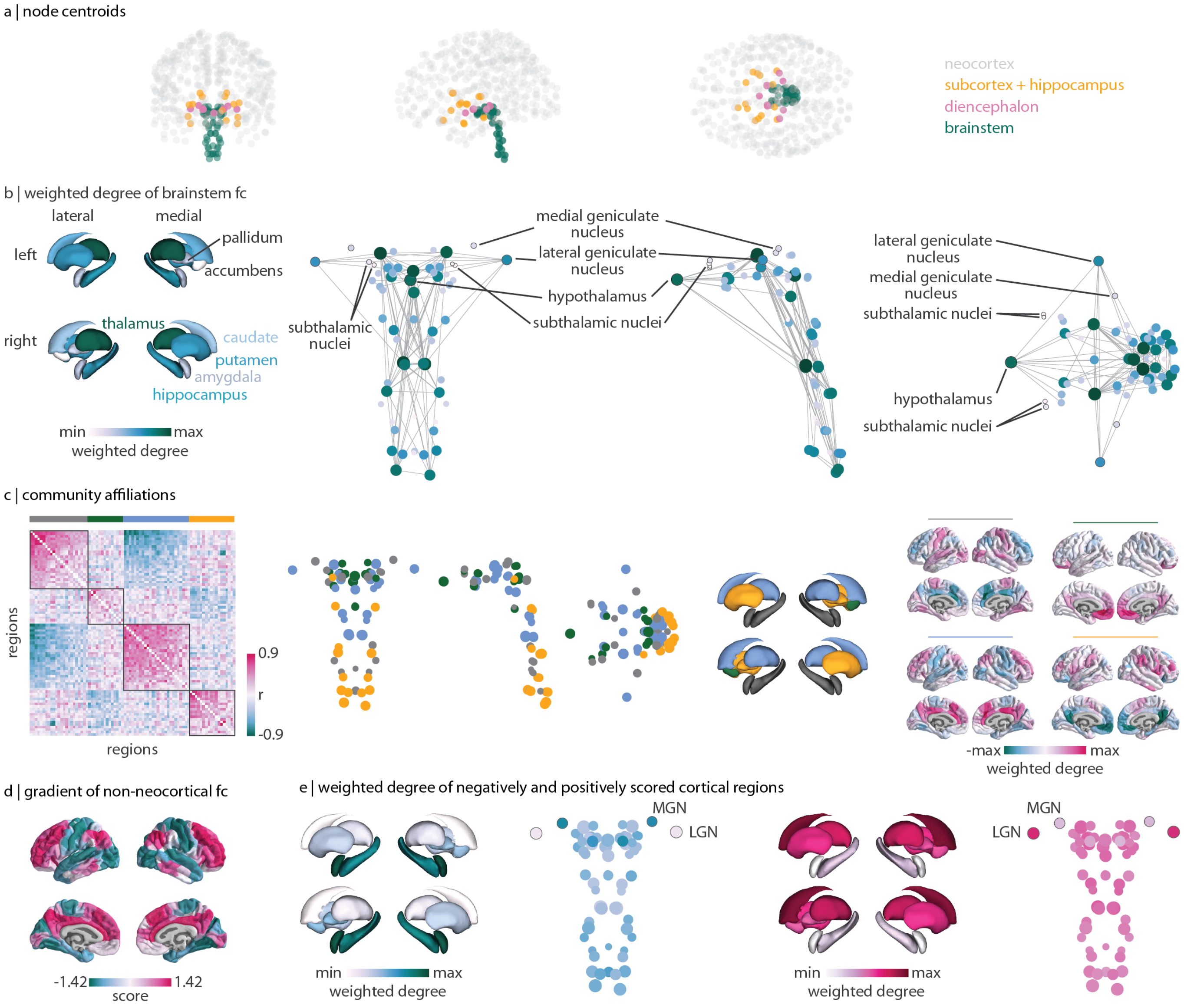


Figure S13. **Extending analyses to subcortical and diencephalic structures** | Functional images were also acquired for the 14 bilateral FreeSurfer subcortical structures (caudate, putamen, pallidum, nucleus accumbens, thalamus, amygdala, hippocampus (not technically subcortex but allocortex)) as well as 8 bilateral diencephalic structures from the Brainstem Navigator (lateral geniculate nucleus, medial geniculate nucleus, subthalamic nuclei subregions 1 & 2). (a) For each brain region, the centroid coordinate is plotted with colours indicating structure. Grey: 400 neocortical structures; yellow: 14 FreeSurfer subcortical structures (including hippocampus); pink: 8 Brainstem Navigator diencephalic regions; green: 58 Brainstem Navigator brainstem nuclei. (b) Left: FreeSurfer subcortical plot of weighted degree of brainstem functional connectivity, representing how much each FreeSurfer subcortical parcel is connected with the brainstem. Each structure is labeled. Right: Brainstem Navigator brainstem and diencephalic centroid coordinates coloured according to their weighted degree of brainstem functional connectivity, representing how much each nucleus is connected with the 58 brainstem structures. The 8 diencephalic nuclei are labeled. (c) Left: region $\times$ region similarity matrix representing how similarly two non-neocortical (i.e. brainstem, subcortical, or diencephalic) regions are functionally connected with the cortex. Outlines are placed around the identified communities. Middle: point brain plot of community assignments for brainstem and diencephalic nuclei and FreeSurfer surface plot of community assignments for FreeSurfer subcortical regions. Right: cortical weighted degree patterns are calculated as the sum of a cortical region's functional connectivity with all brainstem nuclei within a specific community, and are shown for all four communities (labeled by the colour of the bar on top of the cortical surfaces). These maps represent how each brainstem community is connected with the cortex. (d) Coritcal surface plot of the first gradient from diffusion map embedding of how similarly cortical regions are functionally connected with non-neocortical regions. (e) Weighted degree patterns for FreeSurfer subcortical parcels as well as brainstem and diencephalic Brainstem Navigator nuclei, calculated as the sum of a region's functional connectivity with all negatively- (left) or positively- (right) scored regions of the cortical gradient shown on in panel (d). FreeSurfer subcortical structures were plotted using the `enigmatoolbox`[108].

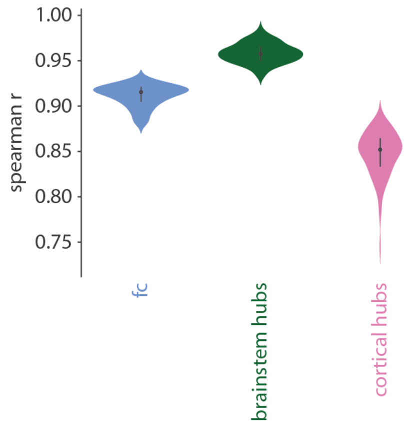


Figure S14. **Split-half analysis | **The 20 participants included in the present study were randomly divided into two groups of 10 ($N = 100$ repetitions). Group-average functional connectivity, brainstem-to-cortex weighted degree patterns, and cortex-to-brainstem weighted degree patterns were recalculated within these groups are correlated. Violin plots estimate a kernel density on the underlying data. The circle indicates the median and the vertical line indicates the quartiles of the distribution.

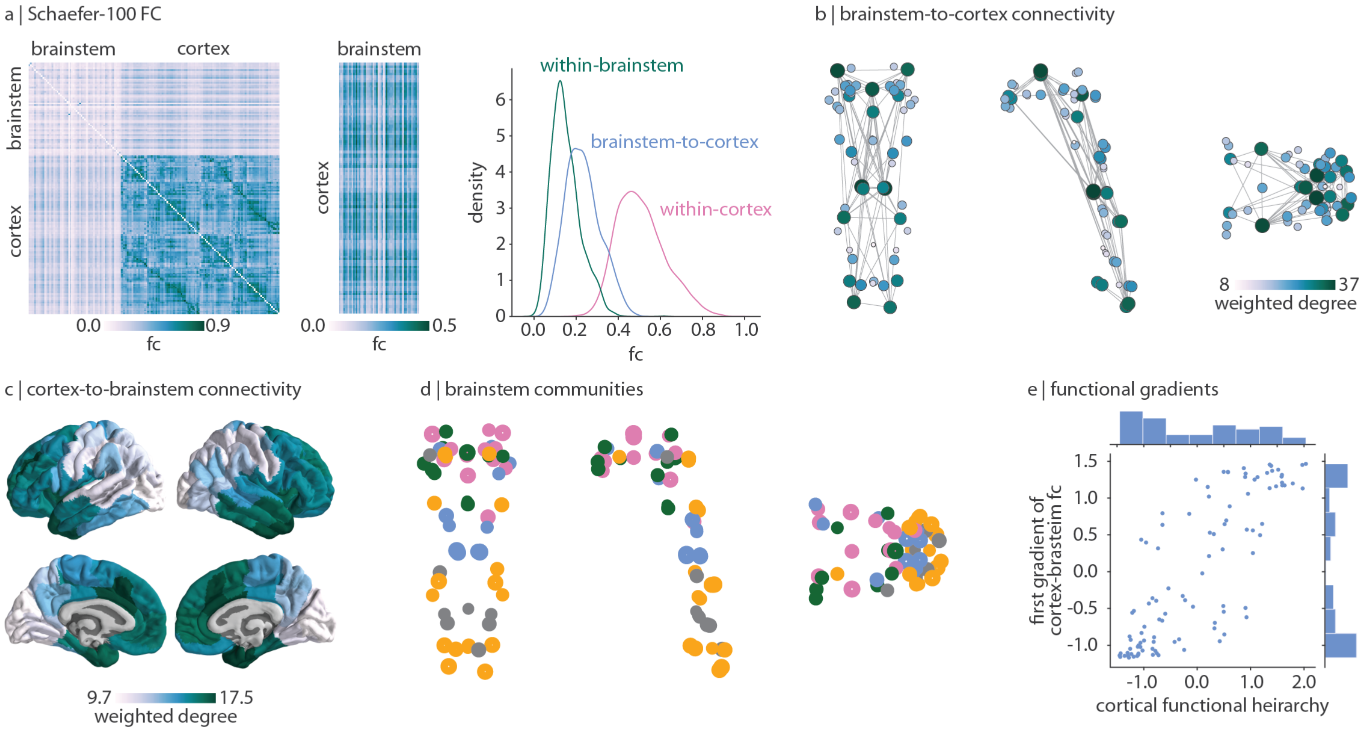


Figure S15. **Replication using 100 cortical regions | **Analyses were repeated using the 100-region Schaefer parcellation[16]. (a) Functional connectivity and functional connectivity density distributions. (b) Brainstem-to-cortex weighted degree. (c) Cortex-to-brainstem weighted degree. (d) Community affiliations of brainstem nodes under identical parameters as shown in Fig. 3. (e) Correlation between the first gradient of cortex-to-brainstem functional connectivity and the cortical functional hierarchy.

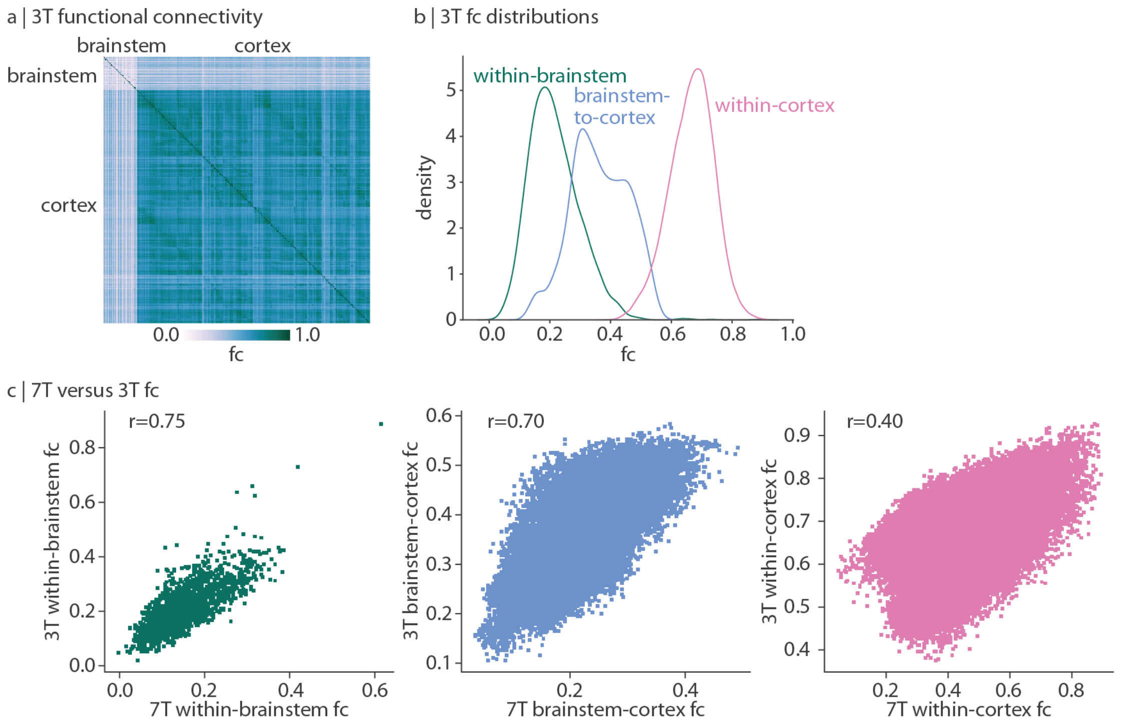


Figure S16. **Replication using 3 Tesla fMRI data |** Analyses were repeated using 3 Tesla fMRI data acquired in the same 20 participants, under the Schaefer-400 parcellation[16]. (a) Functional connectivity. (b) Functional connectivity density distributions. (c) Spearman correlations between 7 Tesla functional connectivity data used in the main analyses and 3 Tesla functional connectivity replication data.

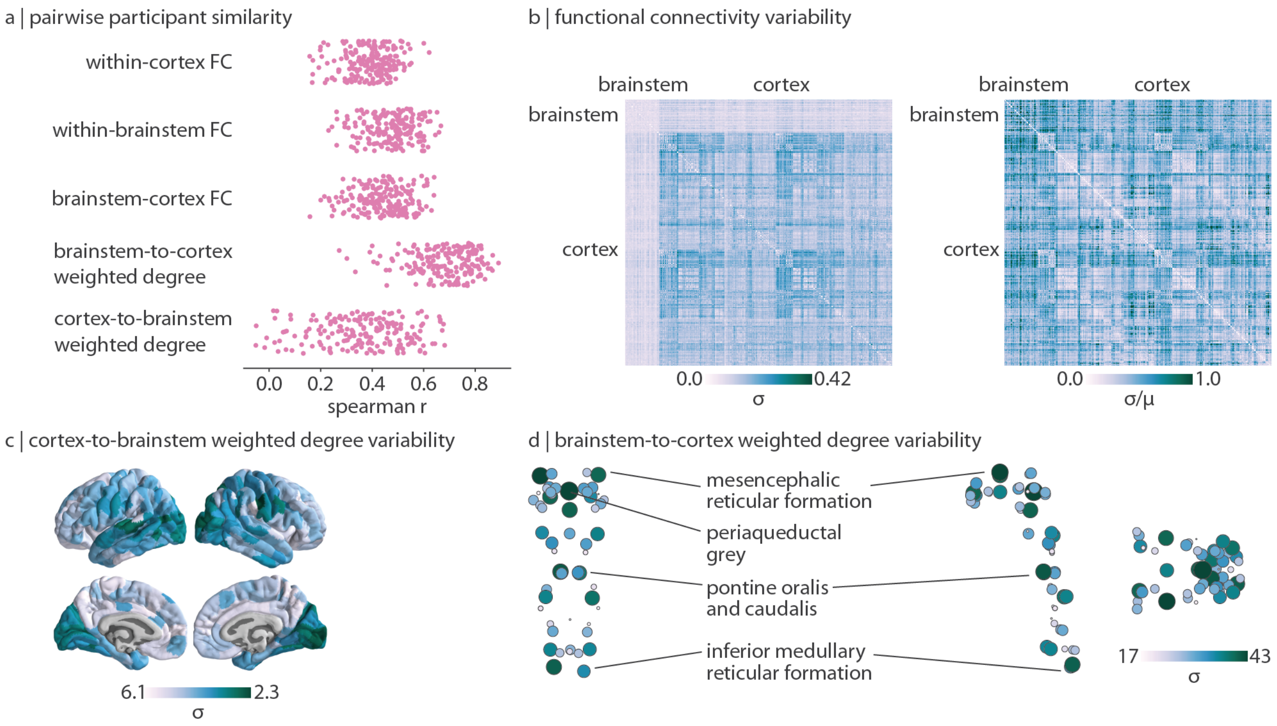


Figure S17. **Individual variability |** We compare functional connectivity and hubness across individual participants. (a) For every pair of participants, we correlate (Spearman $r$) their (1) vectorized upper triangular within-cortex FC, (2) vectorized upper-triangular within-brainstem FC, (3) vectorized brainstem-cortex FC , (4) brainstem-to-cortex weighted degree pattern (mean shown in Fig. 2a), (5) cortex-to-brainstem weighted degree pattern (mean shown in Fig. 2b). (b) Left: standard deviation of functional connectivity across participants. Right: coefficient of variation (standard deviation normalized by mean) of functional connectivity across participants. (c) Standard deviation of cortex-to-brainstem weighted degree across participants. (d) Standard deviation of brainstem-to-cortex weighted degree across participants.

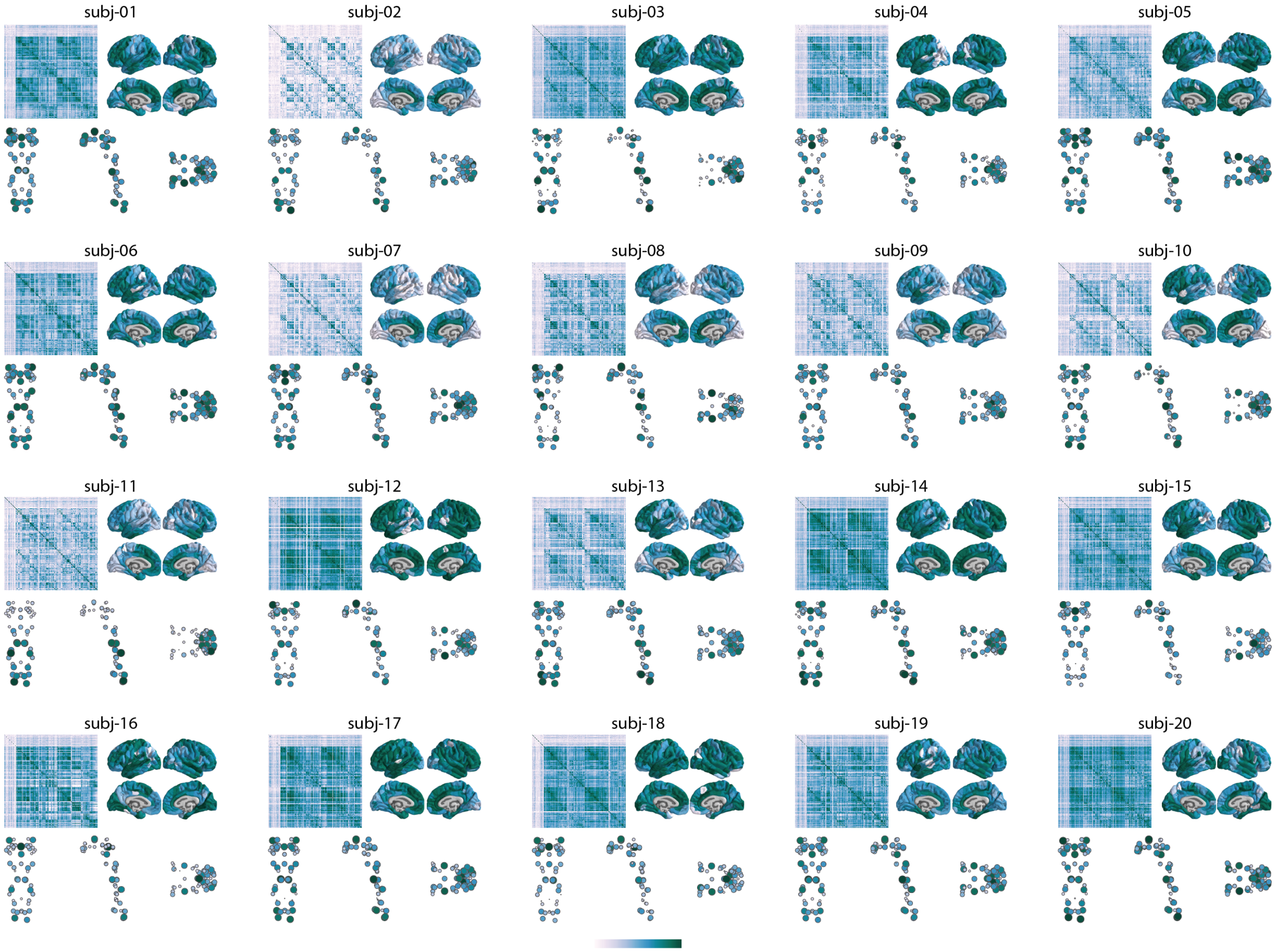


Figure S18. **Individual data |** For each participant (subj-01–subj-20), we show their functional connectivity matrix (mean shown in Fig. 1c), their cortex-to-brainstem weighted degree pattern (mean shown in Fig. 2b), and their brainstem-to-cortex weighted degree pattern (mean shown in Fig. 2a). For functional connectivity matrices, colourbar limits are $[0, 0.9]$. For weighted degree patterns, colourbar limits are the minimum and maximum weighted degree for each participant.

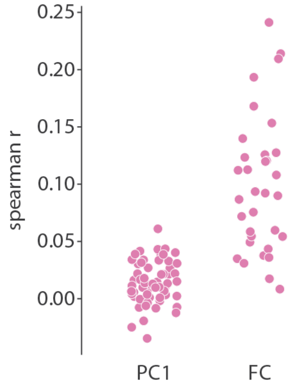


Figure S19. **Correlating brainstem and 4th ventricle signal** | Brainstem fMRI preprocessing includes a CSF noise correction step in which the first five principal components of the signal from brainstem-surrounding CSF (including the 4th ventricle, cerebral aqueduct, and lower part of the 3rd ventricle) are regressed from brainstem time-series. The stripplot on the left shows the Spearman correlation between each brainstem nucleus' time-series and the first principal component regressor, averaged across participants. The stripplot on the right shows the Spearman correlation between brainstem nucleus time-series and 4th ventricle time-series, averaged across participants. Each point is a brainstem nucleus. Note that the right-hand stripplot only includes left hemisphere and midline nuclei ($n = 33$) because voxel-wise FC was not calculated for right hemisphere brainstem nuclei.

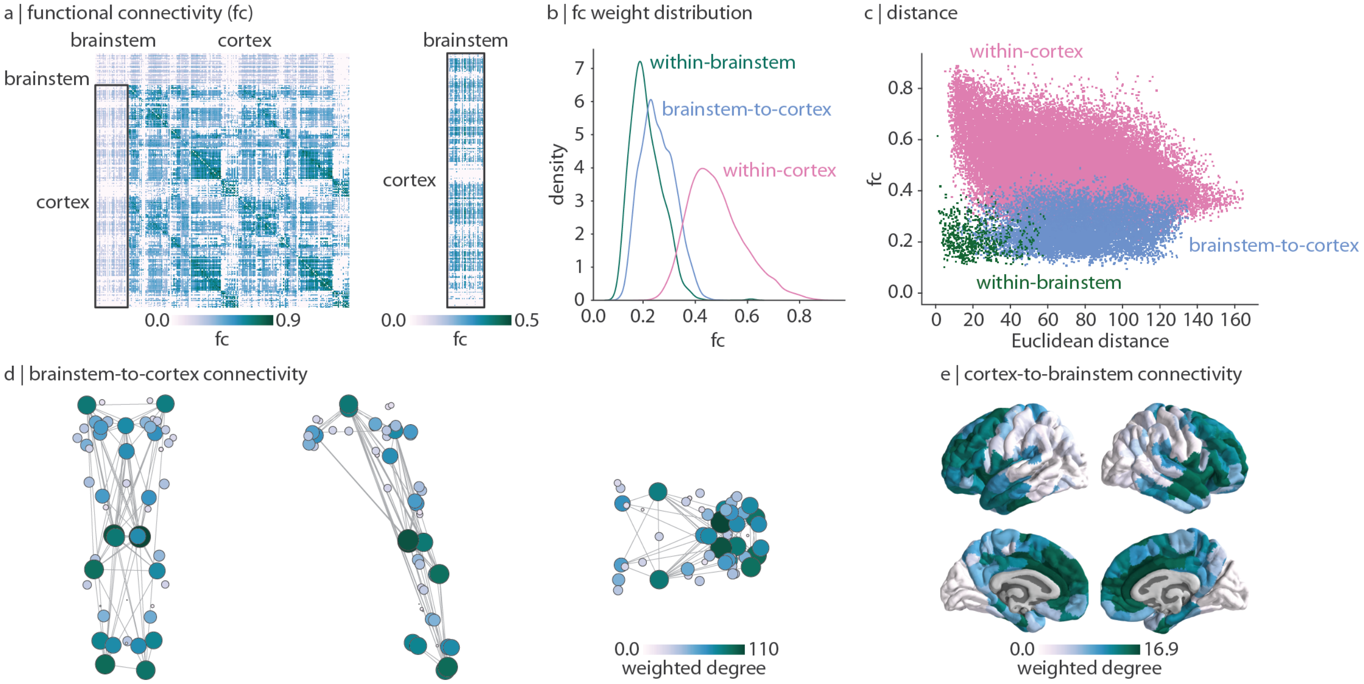


Figure S20. **Replication by thresholding functional connectivity** | A thresholded functional connectivity matrix was constructed (see *Methods* for details) with $53.65\%$ remaining connections. (a) Left: the full 458 $\times$ 458 thresholded functional connectivity matrix. Right: 400 cortical regions $\times$ 58 brainstem nuclei thresholded functional connectivity. (b) Density distributions of functional connectivity within brainstem (green), between brainstem and cortex (blue), and within cortex (pink). (c) Scatter plot of functional connectivity between regions as a function of Euclidean distance between parcel centroids. (d) Brainstem-to-cortex weight degree, calculated as the sum of a brainstem nucleus' functional connectivity across all cortical regions. Spearman correlation of brainstem-to-cortex weighted degree using thresholded and non-thresholded functional connectivity is $r = 0.96$, $p = 2 \times 10^{-32}$ (two-sided Spearman's $r$). Node size and colour reflect weighted degree, and edges are plotted for the 5% strongest functional connections within the brainstem. (d) Cortex-to-brainstem weighted degree, calculated as the sum of a cortical region's functional connectivity across all brainstem nuclei. Spearman correlation of cortex-to-brainstem weighted degree using thresholded and non-thresholded functional connectivity is $r = 0.93$, $p = 9 \times 10^{-171}$ (two-sided Spearman's $r$).

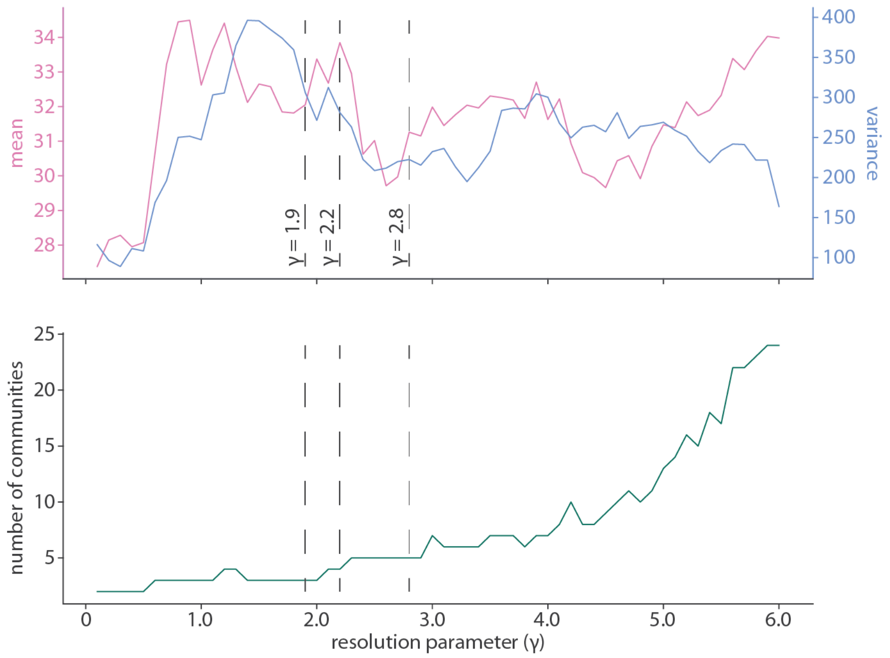


Figure S21. **Community detection performance across resolution parameter $\gamma$ |** Top: mean and variance of the z-scored Rand index across 250 repetitions of the Louvain community detection algorithm at each $\gamma$ (for $\gamma \in [0.1, 6.0]$). Community detection solutions are considered better quality (i.e. more stable) when the mean of the z-scored Rand index is high and the variance is low. Dashed vertical lines are placed at values of $\gamma$ where the community detection solution is shown in the text ($\gamma = 1.9$ shown in Fig. S5, $\gamma = 2.2$ shown in Fig. S6, and $\gamma = 2.8$ shown in Fig. 3). Bottom: the number of communities identified by the algorithm across values of $\gamma$.

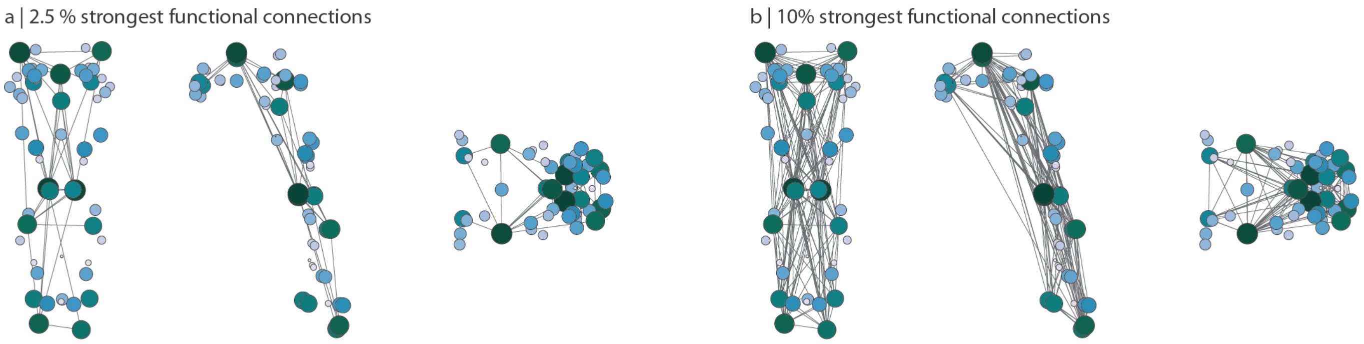


Figure S22. **Strongest functional connections within the brainstem |** In Fig. 2a we plot brainstem-to-cortex weighted degree and edges for the strongest 5% functional connections within the cortex. Here we show (a) the 2.5% strongest functional connections, and (b) the 10% strongest functional connections within the cortex. Node size and colour reflect weighted degree and are identical to those in Fig. 2a.

| | grey | green | blue | yellow |
|---|---|---|---|---|
| brainstem | median raphe nucleus* | periaqueductal grey* | dorsal raphe* | subcoeruleus (L) |
| | raphe magnus* | paramedian raphe nucleus* | caudal-rostral linear raphe* | inferior olivary nucleus (LR) |
| | raphe obscurus* | substantia nigra: pars reticulata (L) | substantia nigra: pars compacta (R) | microcellular tegmental nucleus/parabigeminal nucleus (L) |
| | raphe pallidus* | red nucleus: subregion 1 (LR) | substantial nigra: pars reticulata (R) | lateral parabrachial nucleus (LR) |
| | substantia nigra: pars compacta (L) | microcellular tegmental nucleus/parabigeminal nucleus (R) | ventral tegmental area/ parabrachial pigmented nucleus complex (LR) | viscero-sensory-motor nuclei complex (LR) |
| | red nucleus: subregion 2 (LR) | cuneiform (LR) | medial parabrachial nucleus (LR) | inferior medullary reticular formation (LR) |
| | parvicellular reticular nucleus: alpha part (LR) | superior colliculus (LR) | pontine reticular nucleus: pontis oralis and caudalis (LR) | vestibular nucleus (LR) |
| | superior olivary complex (LR) | | mesencephalic reticular formation (LR) | superior medullary reticular formation (LR) |
| | inferior colliculus (LR) | | pedunculotegmental nucleus (LR) | |
| | subcoeruleus (R) | | isthmic reticular formation (LR) | |
| | | | laterodorsal tegmental nucleus/central grey of the rhomboencephalon (LR) | |
| | | | locus coeruleus (LR) | |
| diencephalon | subthalamic nucleus: subregion 1 (LR) | subthalamic nucleus: subregion 2 (L) | lateral geniculate nucleus (LR) | |
| | subthalamic nucleus: subregion 2 (R) | hypothalamus | | |
| | medial geniculate nucleus (LR) | | | |
| subcortex | amygdala (LR) | accumbens (LR) | caudate (LR) | pallidum (LR) |
| | hippocampus (LR) | | thalamus (LR) | putamen (LR) |

TABLE S1. **Brainstem, diencephalon, and subcortex community assignments |** Brainstem ($n = 58$), diencephalon ($n = 9$), and subcortical ($n = 14$) regions within each of the four communities shown in Fig. S13. Column names refer to the colour of the community, as shown in Fig. S13. Asterisks indicates a midline nucleus. L/R refers to the hemisphere of bilateral nuclei.

| Receptor/ transporter | Neurotransmitter | Tracer | Measure | $N$ | References |
|---|---|---|---|---|---|
| $D_2$ | dopamine | [$^{11}$C]FLB-457 | $BP_{ND}$ | 55 (26) | [122–126] |
| DAT | dopamine | [$^{18}$F]FE-PE2I | $BP_{ND}$ | 6 (6) | [127] |
| NET | norepinephrine | [$^{11}$C]MRB | $BP_{ND}$ | 77 (50) | [128–131] |
| 5-$HT_{1A}$ | serotonin | [$^{11}$C]CUMI-101 | $BP_{ND}$ | 8 (3) | [132] |
| 5-$HT_{1B}$ | serotonin | [$^{11}$C]P943 | $BP_{ND}$ | 23 (15) | [133–139] |
| 5-$HT_{2A}$ | serotonin | [$^{11}$C]Cimbi-36 | $B_{max}$ | 29 (15) | [132] |
| 5-$HT_4$ | serotonin | [$^{11}$C]SB207145 | $B_{max}$ | 59 (41) | [132] |
| 5-$HT_6$ | serotonin | [$^{11}$C]GSK215083 | $BP_{ND}$ | 30 (30) | [140,141] |
| 5-HTT | serotonin | [$^{11}$C]DASB | $B_{max}$ | 100 (29) | [132] |
| $\alpha_4\beta_2$ | acetylcholine | [$^{18}$F]flubatine | $V_T$ | 30 (20) | [142,143] |
| $M_1$ | acetylcholine | [$^{11}$C]LSN3172176 | $BP_{ND}$ | 24 (13) | [144] |
| VAChT | acetylcholine | [$^{18}$F]FEOBV | SUVR | 18 (5) | [145] |
| $mGluR_5$ | glutamate | [$^{11}$C]ABP688 | $BP_{ND}$ | 28 (15) | [146] |
| $GABA_{A/BZ}$ | GABA | [$^{11}$C]flumazenil | $B_{max}$ | 16 (7) | [147] |
| $H_3$ | histamine | [$^{11}$C]GSK189254 | $V_T$ | 8 (7) | [148] |
| $CB_1$ | cannabinoid | [$^{11}$C]OMAR | $V_T$ | 77 (49) | [149–152] |
| MOR | opioid | [$^{11}$C]carfentanil | $BP_{ND}$ | 39 (19) | [153] |

TABLE S2. **Neurotransmitter receptors and transporters** | $BP_{ND}$ = non-displaceable binding potential; $V_T$ = tracer distribution volume; $B_{max}$ = density (pmol/ml) converted from binding potential (5-HT) or distributional volume (GABA) using autoradiography-derived densities; SUVR = standard uptake value ratio. Values in parentheses (under $N$) indicate number of males.
