# Supplementary material for: Integrating brainstem and cortical functional architectures
Source: Nat Neurosci. 2024 Oct 16;27(12):2500–11. doi: 10.1038/s41593-024-01787-0 (PMC11614745; doi:10.1038/s41593-024-01787-0)
Supplement: Supplementary file 2 — Reporting Summary [file 41593_2024_1787_MOESM2_ESM.pdf]

nature portfolio

Corresponding author(s): Bratislav Misic

Last updated by author(s): Aug 12, 2024

Reporting Summary

Nature Portfolio wishes to improve the reproducibility of the work that we publish. This form provides structure for consistency and transparency in reporting. For further information on Nature Portfolio policies, see our Editorial Policies and the Editorial Policy Checklist.

Statistics

For all statistical analyses, confirm that the following items are present in the figure legend, table legend, main text, or Methods section.

n/a	Confirmed	
☐	☒	The exact sample size (*n*) for each experimental group/condition, given as a discrete number and unit of measurement
☐	☒	A statement on whether measurements were taken from distinct samples or whether the same sample was measured repeatedly
☐	☒	The statistical test(s) used AND whether they are one- or two-sided<br>*Only common tests should be described solely by name; describe more complex techniques in the Methods section.*
☐	☒	A description of all covariates tested
☐	☒	A description of any assumptions or corrections, such as tests of normality and adjustment for multiple comparisons
☐	☒	A full description of the statistical parameters including central tendency (e.g. means) or other basic estimates (e.g. regression coefficient) AND variation (e.g. standard deviation) or associated estimates of uncertainty (e.g. confidence intervals)
☐	☒	For null hypothesis testing, the test statistic (e.g. *F*, *t*, *r*) with confidence intervals, effect sizes, degrees of freedom and *P* value noted<br>*Give P values as exact values whenever suitable.*
☒	☐	For Bayesian analysis, information on the choice of priors and Markov chain Monte Carlo settings
☒	☐	For hierarchical and complex designs, identification of the appropriate level for tests and full reporting of outcomes
☐	☒	Estimates of effect sizes (e.g. Cohen's *d*, Pearson's *r*), indicating how they were calculated

Our web collection on statistics for biologists contains articles on many of the points above.

Software and code

Policy information about availability of computer code

Data collection	MEG data was processed using the open software toolbox Brainstorm v220420.<br>Neurosynth meta-analytic maps were fetched using neurosynth v0.3<br>Brainstem data were previously collected and were not specifically collected for the purposes of this study.<br>The Connectome Mapper toolkit release-1.2.0 was used for the spatial nulls.
Data analysis	Python v3.8.10 was used for running scripts and Brainstem Navigator v0.9 was used for identifying brainstem nuclei.<br>A full list of packages used can be found at https://github.com/netneurolab/hansen_brainstemfc/blob/main/environment.yml

For manuscripts utilizing custom algorithms or software that are central to the research but not yet described in published literature, software must be made available to editors and reviewers. We strongly encourage code deposition in a community repository (e.g. GitHub). See the Nature Portfolio guidelines for submitting code & software for further information.

Data

Policy information about availability of data

All manuscripts must include a data availability statement. This statement should provide the following information, where applicable:

- Accession codes, unique identifiers, or web links for publicly available datasets
- A description of any restrictions on data availability
- For clinical datasets or third party data, please ensure that the statement adheres to our policy

All pre-processed data used to perform the analyses are available at https://github.com/netneurolab/hansen_brainstemfc. MEG power spectral data and neurotransmitter receptor/transporter data are available in neuromaps (https://github.com/netneurolab/neuromaps). Neurosynth data is available at https://neurosynth.org/ and the Cognitive Atlas is available at https://www.cognitiveatlas.org/.

Research involving human participants, their data, or biological material

Policy information about studies with human participants or human data. See also policy information about sex, gender (identity/presentation), and sexual orientation and race, ethnicity and racism.

Reporting on sex and gender	Only group-averaged brainstem functional connectivity was analyzed. The group consists of an equal number of males and females. Gender was not considered in the analyses.
Reporting on race, ethnicity, or other socially relevant groupings	Only group-averaged brainstem functional connectivity was analyzed. Data were not grouped by race, ethnicity, or other socially relevant groupings.
Population characteristics	20 healthy subjects volunteered for functional imaging: 10 males, 10 females, age 29.5 +/- 1.1 years.
Recruitment	Subjects were not recruited for the present study but for a previous study (https://www.sciencedirect.com/science/article/pii/S1053811922000544#sec0002).
Ethics oversight	The original study protocol (not done for the purposes of the present study but previously in 2022) was approved by the Massachusetts General Hospital Institutional Review Board. Informed consent was obtained by participants.

Note that full information on the approval of the study protocol must also be provided in the manuscript.

Field-specific reporting

Please select the one below that is the best fit for your research. If you are not sure, read the appropriate sections before making your selection.

☒ Life sciences ☐ Behavioural & social sciences ☐ Ecological, evolutionary & environmental sciences

For a reference copy of the document with all sections, see nature.com/documents/nr-reporting-summary-flat.pdf

Life sciences study design

All studies must disclose on these points even when the disclosure is negative.

Sample size	20 healthy subjects (10 males and 10 females; age 29.5 ± 1.1 years) were recruited to participate in two functional MRI sessions. No predetermined sample size was generated. Sample size in a previous paper (Bianciardi et al 2016 Magn Reson Mater Phy) was 12 and some nuclei showed suboptimal connectivity. Sample size was therefore increased to 20 in Cauzzo & Singh et al 2022 NeuroImage and Singh & Cauzzo et al 2022 NeuroImage, and scan duration tripled, to optimize stability of connectivity across subjects. We confirm that sample size is sufficient by running a split-half analysis and ensuring group-average FC of two 10-subject subsets are highly correlated.
Data exclusions	No data was excluded.
Replication	Images were acquired at 7 Tesla and replicated at 3 Tesla. Furthermore, analyses were performed at the resolution of 400 cortical parcels (Schaefer-400) and replicated at the resolution of 100 cortical parcels (Schaefer-100). A split-half resampling analysis was performed to ensure findings are robust to sample. Finally, analyses were replicated in the subcortex.
Randomization	No experimental groups exist.
Blinding	No experimental groups exist and therefore blinding is not relevant to the study.

Reporting for specific materials, systems and methods

We require information from authors about some types of materials, experimental systems and methods used in many studies. Here, indicate whether each material, system or method listed is relevant to your study. If you are not sure if a list item applies to your research, read the appropriate section before selecting a response.

Materials & experimental systems

n/a	Involved in the study
☒	☐ Antibodies
☒	☐ Eukaryotic cell lines
☒	☐ Palaeontology and archaeology
☒	☐ Animals and other organisms
☒	☐ Clinical data
☒	☐ Dual use research of concern
☒	☐ Plants

Methods

n/a	Involved in the study
☒	☐ ChIP-seq
☒	☐ Flow cytometry
☐	☒ MRI-based neuroimaging

Plants

Seed stocks: n/a

Novel plant genotypes: n/a

Authentication: n/a

Magnetic resonance imaging

Experimental design

Design type: resting-state

Design specifications: 3 resting-state runs were acquired at 7 Tesla (acquisition time per run = 10:07 min)
1 resting-state run was acquired at 3 Tesla (acquisition time = 9:06 min)

Behavioral performance measures: No behavioural measures were acquired.

Acquisition

Imaging type(s): functional MRI

Field strength: 7 Tesla and 3 Tesla

Sequence & imaging parameters: At 7 Tesla:
A custom-built 32-channel receive coil and volume transmit coil was used at 7 Tesla. For each subject, three runs of 7 Tesla functional gradient-echo echo-planar images (EPIs) were acquired with the following parameters: isotropic voxel size = 1.1 mm, matrix size = 180 × 240, GRAPPA factor = 3, nominal echo-spacing = 0.82 ms, bandwidth = 1488 Hz/Px, N. slices = 123, slice orientation = sagittal, slice-acquisition order = interleaved, echo time (TE) = 32 ms, repetition time (TR) = 2.5 s, flip angle (FA) = 75°, simultaneous-multi-slice factor = 3, N. repetitions = 210, phase-encoding direction = anterior-posterior, acquisition-time = 10'07".

At 3 Tesla:
To assess the connectivity reproducibility using 3 Tesla MRI, on the same subjects, we acquired one run of conventional functional gradient-echo EPIs (isotropic voxel size = 2.5 mm, matrix size = 215 × 215, GRAPPA factor = 2, nominal echo-spacing = 0.5 ms, readout bandwidth = 2420 Hz/Px, N. slices = 64, slice orientation = transversal, slice-acquisition order = interleaved, TE = 30 ms, TR = 3.5 s, FA = 85°, N. repetitions = 150, phase-encoding direction = anterior-posterior, acquisition time = 9'06") and a fieldmap (isotropic voxel size = 2.5 mm, FOV = 215 × 215, bandwidth = 300 Hz/Px, N. slices = 128, slice orientation = sagittal, slice-acquisition order = interleaved, TE1 = 4.92 ms, TE2 = 7.38 ms, TR = 849.0 ms, FA = 85°, acquisition time = 2'19", phase-encoding direction = anterior-posterior). Note that, for the purposes of this study, at 3 Tesla a conventional fMRI sequence was used, and the additional 3 Tesla Connectom scanner capabilities were not employed. A custom-built 64-channel receive coil and volume transmit coil was used at 3 Tesla

Area of acquisition: Whole brain

Diffusion MRI ☐ Used ☒ Not used

Preprocessing

Preprocessing software	The root mean square across echo times was extracted from each MEMPRAGE image, the output was then rotated to standard orientation ('RPI'). Bias-field correction was applied with SPM (Frackowiak et al., 1997) tools, then we used FSL routines to extract the brain and crop the image (FMRIB Software Library, FSL 5.0.7, Oxford, UK). Brain parcellations were generated on the MEMPRAGE with Freesurfer (Destrieux et al., 2010) to obtain cortical and subcortical targets. The preprocessed MEMPRAGEs were then iteratively aligned and averaged to build a group-based optimal template with the use of the Advanced Normalization Tool (ANTs, Philadelphia, PA, United States).
Normalization	Images were aligned to the MNI-152 1mm template using ANTs through an affine transformation and a nonlinear warp.
Normalization template	MNI152 1mm.
Noise and artifact removal	Physiological noise correction was done in each resting state fMRI run using custom-built Matlab function of RETROICOR \citep{glover2000magnresonmed} adapted to the slice acquisition sequence. Functional images were then slice-time corrected, reoriented to standard orientation, and coregistered to the MEMPRAGE image. Coregistration was implemented in AFNI using a two-step procedure made of an affine coregistration and a boundary-based (edge enhancing) nonlinear coregistration \citep{cox1996computbiomedres}. Next, six rigid-body motion time-series nuisance regressors, a regressor describing respiratory volume per unit time convolved with a respiration response function \citep{birn2008neuroimage}, a regressor describing heart rate convolved with a cardiac response function \citep{chang2009neuroimage}, and five regressors modeling the signal in cerebrospinal fluid (CSF), extracted using PCA on a mask of the ventricles, were regressed from the fMRI time-series. Cleaned data were scaled to percent signal change by dividing by the temporal signal mean, multiplying by 100, and bandpass filtering between 0.01--0.1 Hz. Finally, any residual temporal mean was removed and the three runs were concatenated.
Volume censoring	n/a

Statistical modeling & inference

Model type and settings	Functional connectivity is defined as the Pearson's correlation between time-series of pairs of regions.
Effect(s) tested	No effects were tested.

Specify type of analysis: ☒ Whole brain ☐ ROI-based ☐ Both

Statistic type for inference (See Eklund et al. 2016)	Voxel-wise.
Correction	n/a

Models & analysis

n/a	Involved in the study
☐	☒ Functional and/or effective connectivity
☐	☒ Graph analysis
☐	☒ Multivariate modeling or predictive analysis

Functional and/or effective connectivity	Pearson's correlation.
Graph analysis	Fully connected networks were derived from the functional connectomes. Weighted degree is defined as a node's summed FC with all other nodes.
Multivariate modeling and predictive analysis	In Figure 4 a multiple linear regression model was fit where independent variables are cortical receptor densities and the dependent variable is a cortical FC pattern associated with a specific brainstem module. Adjusted R-squared are shown.<br>In Figure 5, diffusion map embedding is applied to extract a principal gradient of cortex-brainstem functional connectivity.
